# Chimeric Antigen Receptor T Cell Immunotherapy for Autoimmune Rheumatic Disorders: Where Are We Now?

**DOI:** 10.3390/cells14161242

**Published:** 2025-08-12

**Authors:** Panagiota Anyfanti, Paschalis Evangelidis, Nikolaos Kotsiou, Anna Papakonstantinou, Ioannis Eftychidis, Ioanna Sakellari, Theodoros Dimitroulas, Eleni Gavriilaki

**Affiliations:** 1 Third Department of Internal Medicine, Papageorgiou Hospital, Aristotle University of Thessaloniki, 54124 Thessaloniki, Greece; panyfanti@auth.gr; 2 Second Propaedeutic Department of Internal Medicine, Hippocration Hospital, Aristotle University of Thessaloniki, 54642 Thessaloniki, Greece; pascevan@auth.gr (P.E.); kotsiounikolaos@gmail.com (N.K.);; 3 Medical School, Aristotle University of Thessaloniki, 54636 Thessaloniki, Greece; 4 Hematology Department, BMT Unit, General Hospital “George Papanicolaou”, 57010 Thessaloniki, Greece; ioannamarilena@gmail.com; 5 Fourth Department of Internal Medicine, Hippocration Hospital, Aristotle University of Thessaloniki, 54124 Thessaloniki, Greece

**Keywords:** autoimmune rheumatic disorders, BCMA, CAR-T cell therapy, CD19, immunotherapy, systemic lupus erythematosus

## Abstract

Chimeric antigen receptor (CAR) T cell immunotherapy has changed the landscape of B cell hematological malignancies’ management, while it has recently shown promising results in the treatment of refractory autoimmune rheumatic disorders (ARDs). Targeting B cell antigens such as CD19 and BCMA, CAR-T cell therapy can induce sustained remission by the elimination of autoreactive B cell populations resistant to the standard of care treatment options. Clinical data from case reports and small case series demonstrate profound clinical responses in ARDs, including systemic lupus erythematosus (SLE), systemic sclerosis (SSc), idiopathic inflammatory myopathies (IIMs), rheumatoid arthritis (RA), antiphospholipid syndrome (APS), and primary Sjögren’s syndrome (pSS). Treatment outcomes include reduced disease activity, normalization of serologic markers, improved organ function, and drug-free remission, even after B cell reconstitution. Additionally, toxicities, primarily limited to mild cytokine release syndrome (CRS), were generally manageable with supportive care. Encouraging preliminary results have led to the development of several ongoing clinical trials investigating CAR-T cell therapy across multiple ARDs and patient populations, including pediatric patients. This review summarizes the current clinical experience and provides a comprehensive overview of ongoing clinical trials exploring CAR-T cell immunotherapy for ARDs.

## 1. Introduction

Chimeric antigen receptor T cells (CAR-T cells) are patient-derived T lymphocytes that have been genetically engineered to recognize and eliminate cells expressing a specific surface antigen. Each CAR molecule consists of an extracellular single-chain variable fragment (scFv) that binds the target antigen, a hinge and transmembrane region that anchors the receptor in the T cell membrane, and one or more intracellular signaling domains—typically CD3ζ plus one or two costimulatory modules (e.g., CD28, 4-1BB)—that drive T cell activation, proliferation, and persistence upon antigen engagement [1]. To generate CAR-T cells, peripheral blood mononuclear cells are first collected from the patient by leukapheresis, after which T cells are enriched, activated, and transduced—most often using a lentiviral or retroviral vector encoding the CAR transgene. The modified cells are then expanded ex vivo to reach a therapeutic dose, the patient undergoes a short course of lymphodepleting chemotherapy (LDC) to create “space” and reduce regulatory influences, and finally the CAR-T product is infused back into the patient [1]. Once administered, CAR-T cells traffic to sites of antigen expression, form an immunological synapse with target cells, and unleash cytotoxic mechanisms—perforin/granzyme release and death receptor signaling—to eliminate pathogenic cells, while also proliferating in vivo to maintain long-term surveillance.

Over the past four decades, therapeutic strategies for autoimmune rheumatic diseases have progressed from broad-spectrum immunosuppression and anti-T cell regimens in the 1980s–90s to targeted B cell depletion in the 2000s—most notably with rituximab’s serendipitous success in rheumatoid arthritis, which established anti-CD20 monoclonals as a mainstay of therapy. CD20′s exceptionally high surface density (~150,000 copies per cell) enables efficient antibody binding, complement activation, and cell-mediated cytotoxicity against mature effector and memory B cells. By contrast, CD19-directed CAR-T cells harness a pan-B-lineage target that spans early progenitors through plasmablasts but at a lower antigen density (~28,000 copies per cell). This fivefold difference in effector/target stoichiometry, coupled with the distinct in vivo kinetics and tissue trafficking of a living cellular product versus a soluble antibody, underlies both the deeper reservoir depletion and the heightened risk of on-target, off-tumor cytopenias seen with CAR-T approaches. Appreciating these mechanistic nuances clarifies why CAR-T may achieve more profound, durable remissions in refractory autoimmunity, yet also demands the careful design of LDC and safety switches to balance efficacy with tolerability.

CAR-T cell therapies have remarkably changed the therapeutic outcomes of patients with relapsed or refractory (R/R) B cell malignancies by employing genetically engineered T cells to express CARs, aiming at specific target antigens [1]. CD19 is expressed in normal and malignant B cells during all the developmental stages of the B cell maturation process, making it an excellent candidate as a CAR-T cell target [2]. To date, four anti-CD19 CAR-T cell products have been approved by the Food and Drug Administration (FDA) for R/R B cell hematological malignancies: axicabtagene ciloleucel, brexucabtagene autoleucel, lisocabtagene maraleucel, and tisagenlecleucel [3]. Another target of CARs includes the B cell maturation antigen (BCMA), expressed in the cell membrane of plasma cells [4]. Until today, two CAR-T cell (anti-BCMA) immunotherapy products have been approved for the management of patients with R/R MM: idecabtagene vicleucel and ciltacabtagene autoleucel [5].

Nevertheless, toxicities and other complications might be manifested even within 30–60 min in the post-infusion period in CAR-T cell recipients [6,7]. Cytopenias, infections, and endothelial injury syndromes, including cytokine release syndrome (CRS) and immune effector cell-associated neurotoxicity syndrome (ICANS), are among the most serious complications reported [8]. In contemporary practice, however, these early complications are readily managed, i.e., the prophylactic and on-demand administration of IL-6 receptor antagonists (e.g., tocilizumab), corticosteroids, and supportive measures (such as vasopressors and oxygen supplementation) rapidly attenuate both CRS and ICANS without diminishing anti-target efficacy. Moreover, next-generation CAR designs increasingly incorporate built-in safety switches (e.g., inducible caspase-9), tunable costimulatory domains (CD28 versus 4-1BB) to modulate in vivo expansion kinetics, and small-molecule-gated “on/off” constructs that allow the real-time control of CAR-T activity [3]. These advances mean that, while vigilant monitoring during the first hour after infusion remains essential, the potent and durable remissions achieved in both oncology and autoimmune settings far outweigh the manageable risks posed by early toxicities.

Moreover, reports of cardiovascular toxicity, such as arrhythmias, left ventricular systolic dysfunction, significant hypotension, and subsequent shock, with treatment demanding inotropic support, have been reported [9,10].

ICANS and CRS constitute the major complications of CAR-T cell immunotherapy, observed mainly in the early post-infusion period. According to the American Society for Transplantation and Cellular Therapy (ASTCT) criteria, ICANS can be categorized into four grades (grade 1–4) based on the clinical manifestations of the patient, which include a diverse clinical presentation, ranging from dizziness, tremors, and headaches to delirium, hallucinations, seizures, and cognitive deficits [11,12]. Regarding CRS pathogenesis, an inflammatory response leads to the rapid activation and circulation of CAR-T cells, resulting in high levels of circulating cytokines, such as interleukin (IL-6), and, consequently, life-threatening treatment-related toxicity [13]. Fever is an early sign of CRS, followed by capillary leak, hypoxia, and hypotension. ASTCT has also proposed grading criteria for CRS, which are based on the patient’s clinical condition. Corticosteroids and tocilizumab, an antagonist of the anti-IL-6 receptor, are the standard of care in CRS management [14].

Beyond the several hematological malignancies, the efficacy of CAR-T immunotherapy is also being examined for the treatment of patients with autoimmune disorders, based on the autologous transplantation efficacy in this field. In this review, we aim to summarize the published clinical data regarding the outcomes of CAR-T cell immunotherapy in patients with autoimmune rheumatic disorders. Moreover, we present an overview of the ongoing phase I/II clinical trials in the field, while presenting the molecular insights implicated in the CAR-T cell product’s mechanism of action.

## 2. Burden of Autoimmune Rheumatic Disorders

According to contemporary estimates, autoimmune diseases affect approximately one in ten individuals, with continuously increasing trends over time [15]. Autoimmune rheumatic disorders constitute a group of heterogeneous, rare, multisystem diseases associated with increased morbidity and mortality rates [16]. While clinical manifestations and organ involvement may substantially differ within the wide spectrum of autoimmune rheumatic disorders, including rheumatoid arthritis (RA), systemic lupus erythematosus (SLE), myositis, and systemic sclerosis (SSc), they represent debilitating, potentially life-threatening, painful conditions severely compromising health-related quality of life. An excessive self-reactive immune response characterized by loss of tolerance to auto-antigens is a fundamental pathobiological finding mutually encountered in all of these clinical entities [17]. While the precise underlying cellular and molecular mechanisms remain poorly understood, recent mechanistic insights have unfolded the importance of regulatory B cells (Breg cells) as important immunoregulatory factors [18].

The pharmaceutical armamentarium for autoimmune rheumatic disorders has substantially evolved over the recent decades with the introduction of novel therapies, including biologic agents and targeted small molecules. While current antirheumatic medications may slow down the disease progression, they confer no definite treatment, nor do they manage to fully reverse the damage already done; they typically need to be administered lifelong, and they are frequently accompanied by short- and long-term toxicities, including cardiovascular complications [16,19].

Hence, there are presently huge unmet needs for improved therapeutic options tailored to patients with refractory autoimmune rheumatic disorders. In this field, autologous hematopoietic cell transplantation (auto-HCT) has been effectively used for several autoimmune diseases, given the fact that immune system reconstitution can be achieved with this treatment approach. Based on European Bone Marrow Transplantation (EBMT), auto-HCT is included in several treatment algorithms for autoimmune rheumatic disorders, especially for SSc [20]. With the successful use of auto-HCT and the increased burden of disease that people living with rheumatic diseases experience, CAR-T cell immunotherapy might be a potentially novel treatment approach for these patients.

## 3. Clinical Studies on CAR-T Cell Immunotherapy for Autoimmune Rheumatic Disorders

### 3.1. Systemic Lupus Erythematosus (SLE)

SLE is marked by a breakdown of tolerance to nuclear antigens, leading to the generation of pathogenic autoantibodies and subsequent immune-complex-mediated inflammation affecting the kidneys, skin, joints, and multiple other organs [21,22]. Standard B cell-depleting agents such as rituximab and belimumab often produce incomplete or transient remissions, largely owing to the persistence of tissue-resident B cells and long-lived plasma cells that lack CD20 expression [21,22]. In contrast, CD19-targeted CAR-T cells achieve systemic B cell ablation regardless of anatomical location, thereby raising the possibility of durable, drug-free remission. The inaugural report of CAR-T therapy in human SLE was published by Mougiakakos et al. in 2021 [23]. They described a 20-year-old female patient with severe, refractory SLE who presented with class IIIA lupus nephritis (SLE-LN), nephrotic syndrome, pericarditis, pleuritis, malar rash, arthritis, and a prior history of Libman–Sacks endocarditis. Despite aggressive treatment with hydroxychloroquine, high-dose corticosteroids, cyclophosphamide (CYC), mycophenolate mofetil, tacrolimus, belimumab, and rituximab, her disease remained uncontrolled. Following LDC with fludarabine and CYC, she received a single infusion of 1.1 × 10^6^ CD19 CAR-T cells/kg (with a CD4^+^: CD8^+^ ratio of 3:1). Notably, she did not experience significant complications such as CRS, ICANS, or prolonged cytopenias. Her circulating B cells were eliminated for over 44 days, and her anti-dsDNA antibodies and complement levels were normalized. Clinically, she exhibited marked improvement in proteinuria, and her SLE disease activity index (SLEDAI) score declined from 18 to 0 after treatment.

Moreover, Mackensen et al. reported a cohort of five adults (four females, one male; median age: 22 years) who were refractory to multiple immunosuppressive regimens [24]. Each patient received autologous CD19 CAR-T cells (1 × 10^6^ cells/kg) following LDC with fludarabine and CYC. In 3 months post-CAR-T cell infusion, all five patients achieved complete, drug-free remission: the SLEDAI scores declined to zero, anti-dsDNA and antinuclear antibody (ANA) titers normalized, and proteinuria resolved. Additionally, remission persisted without immunosuppressive therapy for a median follow-up of 8 months (range: 1–12 months) even after B cell reconstitution, which occurred at a mean of 110 ± 32 days. Importantly, the re-emergent B cells exhibited a naïve, non-class-switched phenotype. Treatment-related adverse events were minimal, i.e., three patients experienced grade 1 CRS (fever, hypotension), two of whom received tocilizumab; no ICANS or serious infections were observed. Long-term follow-up data extending to 29 months confirmed sustained remission in all participants without further immunosuppression [25].

In another case, a 32-year-old female with refractory SLE, diagnosed during pregnancy and characterized by serositis, nephritis, cytopenias, hypocomplementemia, and suspected central nervous system involvement, received CD19 CAR-T therapy [26]. Before leukapheresis, all immunosuppressants—except for a minimal corticosteroid dose—were tapered. The CAR-T infusion was well tolerated, and within three months, she attained the lupus low-disease activity state (LLDAS) criteria: proteinuria resolved and circulating anti-dsDNA antibodies became undetectable. Notably, her B cells reappeared in peripheral blood within two months, yet there was no disease recurrence. Hagen et al. subsequently extended CAR-T application to severe CNS lupus [27]. Specifically, a 21-year-old male presenting with transverse myelitis and vasculitis (initial SLEDAI: 22) received a single dose of 1 × 10^6^ CD19 CAR-T cells/kg. By week 12, the SLEDAI score was zero, exhibiting full neurologic recovery, while MRI lesions had regressed. CRS and ICANS were absent; anti-dsDNA and interferon-alpha levels became undetectable. This case highlights CAR-T cell penetration into the central nervous system and the durable suppression of neuroinflammation.

In the context of bispecific (dual) CAR-T cells, a 41-year-old woman with a 20-year history of severe, refractory SLE was diagnosed with stage IV DLBCL [28]. After discontinuing the first-line chemotherapy regimen due to intolerance, she received LDC with fludarabine/CYC followed by an infusion of 5.3 × 10^6^ CAR-T cells/kg. These “compound” CARs simultaneously targeted both CD19 and BCMA. In the two-month period following infusion, CAR-T cells expanded in peripheral blood (peaking at ~8% of lymphocytes), while circulating B cells became undetectable by day 198 and only recovered to normal levels 9 months later. Despite the cessation of all other immunosuppression, complement C3 and C4 remained within normal limits, indicating quiescent disease. ANA titers (nuclear, granular, and cytoplasmic patterns) dropped to undetectable levels by 9, 12, and 37 weeks post-CAR-T cell infusion, respectively, accompanied by marked reductions in serum IgG, IgM, and IgA levels. Positron emission tomography and computed tomography (PET-CT) scan at 4 months demonstrated the complete remission of DLBCL, with the disappearance of prior iliac lymphadenopathy and bone marrow plasma cells, as confirmed by flow cytometry. At 23 months post-infusion, the patient remained in clinical remission for both SLE (no clinical or serologic relapse despite the recovery of B cells) and DLBCL without further immunosuppression.

In pediatric patients, He et al. treated two 12-year-old female patients with refractory SLE [29]. The first patient presented with cutaneous ulcers, arthritis, macrophage activation syndrome, and nephrotic-range proteinuria; the other patient had hypertension, pleuritis, hematuria, and biopsy-confirmed class IV SLE-LN. Both underwent fludarabine/CYC LDC, discontinued prior immunosuppression, and received autologous CD19 CAR-T cells. CAR-T expansion peaked at days 7–10, achieving complete B cell aplasia by day 7; while B cells reappeared around day 60 post-infusion. Adverse events were limited to grade 1 CRS (fever) in both patients and transient grade 1 neurotoxicity in one patient, managed with ibuprofen and short-course steroids. Transient hypogammaglobulinemia was treated with intravenous immunoglobulin. By months 4–5, the first patient’s cutaneous lesions and proteinuria resolved, complement C3 was normalized by day 28, anti-dsDNA antibodies became undetectable, and SLEDAI-2K decreased from 12 to 0. In the second patient, pleuritis and hematuria resolved, anti-dsDNA antibodies normalized, and SLEDAI-2K was lowered from 12 to 4; proteinuria dropped from 28 mg/kg/day to 13.6 mg/kg/day, and repeat biopsy showed improved activity but persistent chronic changes regarding SLE-LN. Both discontinued all immunosuppression after CAR-T infusion and maintained clinical improvements at four- and five-month follow-ups, respectively. This preliminary report suggests that low-dose CD19 CAR-T therapy is feasible, well tolerated, and highly effective in pediatric refractory SLE.

Collectively, clinical evidence in SLE indicates that CD19-directed CAR-T cells can result in the reduction in the SLEDAI scores, resolution of proteinuria, normalization of complement levels, and, in some cases, achievement of complete remission. A significant percentage of patients attain steroid- and immunosuppression-free states by month 6. Additionally, B cell recovery, typically occurring around four months, retains a naïve phenotype and permits functional vaccine responses.

### 3.2. Systemic Sclerosis (SSc)

SSc is characterized by autoantibody production against antigens such as topoisomerase I and RNA polymerase III, widespread fibrosis of the skin and internal organs, and microvascular injury [30,31]. B cell dysregulation contributes to disease pathogenesis by generating autoantibodies and secreting cytokines, including IL-6, and transforming growth factor beta (TGF-β) that activate fibroblasts. Bergmann et al. described a 60-year-old male patient with diffuse cutaneous SSc (dcSSc)—manifesting skin, pulmonary, and cardiac fibrosis—and positive anti-RNA polymerase III antibodies [32]. At baseline, 22 months after the first non-Raynaud’s symptom, he exhibited myocardial fibrosis on cardiac magnetic resonance imaging (MRI), interstitial lung disease (ILD) on high-resolution CT, pulmonary hypertension, Raynaud’s phenomenon, and carpal arthritis. Previous therapies (methotrexate 15 mg/week for 3 months and mycophenolate mofetil 2 g/day for 23 months) had failed; CYC was eschewed due to limited efficacy for arthritis and the possible concerns regarding cardiac involvement. All immunosuppression was tapered and discontinued four weeks prior to CAR-T infusion. Autologous T cells were collected via leukapheresis, transduced with a CD19 CAR lentiviral vector (Miltenyi), and expanded on the CliniMACS Prodigy platform. Because of pre-existing kidney disease, LDC doses were reduced by 50% (fludarabine 12.5 mg/m^2^ on days −5 to −3 and CYC 500 mg/m^2^ on day −3). On day 0, he received 1 × 10^6^ CAR-T cells/kg. CAR-T cells expanded from 0.1% of CD3^+^ T cells (0.3 cells/µL) on day 3 to 66.35% of CD3^+^ T cells (1275 cells/µL) by day 9; by day 119, CAR-T cells remained detectable at 1% of T cells. Peripheral B cell depletion was complete by day 7. Clinically, the patient’s modified Rodnan skin score (mRSS) decreased progressively, and his joint inflammation resolved. Pulmonary function stabilized: forced vital capacity (FVC) rose, and right heart catheterization revealed improved hemodynamics. Additionally, skin thickening regressed, and signs of myocardial fibrosis (troponin T levels) stabilized after CAR-T cell infusion. Adverse events were limited to grade 1 CRS (fever), without ICANS, infectious complications, or persistent cytopenias. ANA and anti-RNA polymerase III antibody levels were undetectable by month 3 post-infusion.

Furthermore, Auth et al., in their study, described six patients with severe, diffuse SSc who received 1 × 10^6^ CD19 CAR-T cells/kg following standard LDC (fludarabine/CYC) [33]. Specifically, over a median follow-up of 487 days, none of them experienced predefined events, such as ILD progression, organ failure, or a need for new immunosuppressants. By 6 months, the median American College of Rheumatology Composite Response Index in Systemic Sclerosis (ACR-CRISS) improvement probability reached 100%. Skin scores declined by a median 31% (approximate eight-point mRSS reduction) within 100 days, while high-resolution CT showed a 4% median decrease in the ILD extent—driven by diminished ground-glass opacities—and FVC improved by 195 mL at the last follow-up. Moreover, CRS was mild or absent (one patient grade 0; three patients grade 1; two patients grade 2), while no ICANS events were reported. Nevertheless, one patient required hospitalization for influenza respiratory infection complicated with bacterial superinfection. Analogous to SLE findings, ANA and SSc-specific antibodies declined post-CAR-T therapy.

In their recently published study, Müller et al. included 15 patients with refractory rheumatic autoimmune disorders—8 with SLE, 3 with idiopathic inflammatory myopathies (IIMs), and 4 with SSc—who received autologous CD19 CAR-T cells after LDC [25]. CAR-T cell expansion peaked by day 7, along with complete B cell aplasia. Furthermore, by 3 to 6 months post-infusion, 100% of patients achieved clinical remissions: SLE patients met low-disease activity/remission criteria; IIM patients attained ACR-EULAR major response; and SSc patients showed significant reductions in the EUSTAR activity index and mRSS. Interestingly, these improvements persisted for 12 to 29 months. Serologically, SLE-related autoantibodies normalized; IIM patients’ creatine kinase levels and muscle strength normalized; and SSc patients exhibited reduced skin fibrosis and ILD burden, enabling the discontinuation of glucocorticoids and immunosuppressants by month 6 without relapse. Adverse events were manageable, i.e., 73% of patients experienced primarily grade 1–2 CRS; one patient developed grade 1 ICANS; and infections were mostly mild. Transient hypogammaglobulinemia resolved with short-term intravenous immunoglobulin administration, while long-term immunoglobulin levels remained protective.

In a case report of Merkt et al., a 38-year-old female with anti-Scl-70-positive diffuse SSc and progressive nonspecific interstitial pneumonia was described [34]. In this patient, mycophenolate and nintedanib were ceased before the administration of third-generation CD19 CAR-T cells. Regarding the infusion-related toxicities, only grade 1 CRS occurred. Moreover, CAR-T expansion peaked by day 9, and B cells were fully depleted by day 7. Over an 11-month follow-up, her mRSS steadily improved, pulmonary function markers (FVC, diffusing capacity of the lungs for carbon monoxide (DLCO)] increased, and CT imaging revealed the regression of ground-glass opacities and fibrotic areas in the lungs. Moreover, anti-Scl-70 antibodies, C-reactive protein (CRP), and high-sensitivity troponin T normalized, and Fcγ-receptor-activating immune complexes disappeared, indicating durable immunologic remission. No severe adverse events occurred, and clinical improvements persisted even after reintroducing—and subsequently withdrawing—MMF and nintedanib.

Based on the findings of these studies, CD19 CAR-T therapy in SSc is demonstrated as a rapid inducer of B cell aplasia, while significantly regressing fibrosis (both cutaneous and pulmonary), resulting in durable clinical remission with an acceptable safety profile.

### 3.3. Rheumatoid Arthritis (RA)

RA is characterized by chronic synovial inflammation driven by autoreactive B and T lymphocytes, while anti-cyclic citrullinated peptide (anti-CCP) antibodies constitute a hallmark serologic feature of this clinical entity [19,35]. Szabo et al. reported a 73-year-old male with long-standing, refractory RA who subsequently developed DLBCL after more than a decade of receiving therapy with several immunosuppressive agents, including methotrexate and tocilizumab [36]. The patient was enrolled in a phase II trial of bispecific (dual) anti-CD20/CD19 CAR-T cells (zamtocabtagene autoleucel) primarily for the management of DLBCL. RA medications were discontinued before LDC with fludarabine and CYC, and freshly manufactured, non-cryopreserved anti-CD20/CD19 CAR-T cells were infused. In the early post-infusion period, only grade I CRS (fever) was observed. Additionally, rapid in vivo CAR-T expansion and sustained B cell aplasia occurred by day 180. Over the subsequent months, the rheumatoid factor levels decreased from approximately 1200 IU/mL to 13 IU/mL, and complete, drug-free RA remission was achieved by week 4, persisting through one year of follow-up. Concurrently, his DLBCL progressed from a partial to a complete metabolic response by week 48. Hematologic recovery was uncomplicated aside from transient neutropenia managed with intermittent filgrastim, and immunoglobulin levels remained adequate without intravenous immunoglobulin replacement. This case illustrates that bispecific (dual) anti-CD20/CD19 CAR-T therapy can induce durable, immunosuppression-free remission in both RA and concurrent DLBCL, with an acceptable safety profile.

### 3.4. IIMs

IIMs—including antisynthetase syndrome and immune-mediated necrotizing myopathy (IMNM)—are often mediated by autoantibodies such as anti-Jo-1 and anti-signal recognition particle (anti-SRP), accompanied by severe muscle inflammation and other clinical manifestations, such as ILD [37]. Mueller et al. described a case of refractory anti-Jo-1 antisynthetase syndrome in a 41-year-old male patient [38]. The patient had severe myositis [creatine kinase (CK) > 9000 U/L], ILD, and high-titer anti-Jo-1 antibodies despite prior treatment with corticosteroids, rituximab, tacrolimus, IVIG, and CYC. After LDC with fludarabine and CYC, he received a single infusion of 1 × 10^6^ CD19 CAR-T cells/kg (4-1BBζ costimulatory domain). Grade 1 CRS (fever and transient myalgia with CK spikes) managed with tocilizumab was reported in the early post-infusion period, while no neurotoxicity events occurred. Furthermore, by day 180, CK normalized (13,600 U/L to 102 U/L), anti-anti-Jo-1 titer declined from 331 U/mL to 5 U/mL, and improvement in muscle strength was reported. Additionally, findings of ILD resolved on chest CT, and functional tests (sit-to-stand, 5 km walk) normalized. Peripheral B cells remained undetectable through day 90 and re-emerged with a naïve phenotype. Nevertheless, monthly IVIG administration for transient hypogammaglobulinemia was required while clinical remission persisted through 6 months, allowing the complete discontinuation of immunosuppression.

Pecher and colleagues described a case of a 41-year-old male patient with anti-Jo-1 antisynthetase syndrome refractory to previous treatment lines (rituximab, azathioprine), who received 1.23 × 10^6^ CD19 CAR-T cells/kg after LDC with fludarabine (25 mg/m^2^ days −5 to −3) and CYC (1000 mg/m^2^ day −3) [39]. Moreover, mycophenolate mofetil (2 g/day) was initiated on day 35 to co-target CD8^+^ T cells, which were thought to perpetuate the disease. CAR-T cells expanded without inducing CRS or ICANS, achieving B cell aplasia by day 7. Within weeks, muscle enzymes, including creatine kinase, aspartate and alanine transaminases, and lactate dehydrogenase, CD8^+^ T cell subsets, and proinflammatory cytokines [intefernon-γ (IFN-γ), interleukin-1 (IL-1), IL-6, interleukin-13 (IL-13)] normalized, while anti-Jo-1 antibody levels declined, and a partial recovery was observed in the immunoglobulins levels (IgA 67%, IgG 87%, and IgM 58% of normal levels). Moreover, clinically, muscle strength and pulmonary function improved significantly, while thigh MRI and lung function tests showed regression of inflammatory lesions. It must be underlined that these remissions persisted through 8 months of follow-up, demonstrating that CD19 CAR-T combined with short-term MMF can effectively reset both B and T cell-mediated pathways in refractory antisynthetase syndrome.

Furthermore, Volkov et al. treated a 39-year-old male with refractory anti-SRP-positive IMM in a phase I trial using a fully human 4-1BBζ CD19 CAR-T construct (CABA-201) [40]. CAR-T cells achieved peripheral B cell depletion by day 15, and CK levels normalized by week 8, accompanied by improved muscle strength and endurance. No CRS or ICANS occurred, while immunoglobulin levels remained stable. Autoantibodies against SRP-9, SRP-72, SRP-54, and Ro-52 declined relative to baseline, whereas antibodies against pathogens and vaccine antigens remained unchanged (in comparison to pre-infusion levels). In vitro assays confirmed that the infused product comprised predominantly CD4^+^ effector memory T cells with robust cytolytic activity. Post-infusion dynamics included a peak in CABA-201 expansion on day 15, preceded by an IFN-γ peak on day 8 and increases in interleukin-12 (IL-12) on day 15, underscoring the favorable safety, efficacy, and pharmacodynamic profile of CABA-201 in the first IMNM subject.

### 3.5. Antiphospholipid Syndrome (APS)

A 65-year-old female with longstanding systemic lupus erythematosus (SLE) and triple-positive antiphospholipid syndrome (APS), complicated by recurrent deep vein thrombosis despite long-term anticoagulation with vitamin K antagonists, developed a relapsed aggressive B cell lymphoma [41]. The patient was treated with anti-CD19 CAR-T cell therapy (axicabtagene ciloleucel) following standard LDC (fludarabine and CYC). The patient experienced grade 1 CRS, which was managed effectively with tocilizumab and dexamethasone, and grade 4 ICANS, which resolved following high-dose methylprednisolone administration. At 1-year post-infusion, she remained in complete remission from the lymphoma. Notably, antiphospholipid antibodies—including anticardiolipin IgG/IgM and anti-anti-β2-glycoprotein I IgG/IgM—declined rapidly post-treatment and became undetectable by day 79. Lupus anticoagulant and ANA also converted to negative, reflecting the broad suppression of the autoreactive B cell repertoire. Throughout the 12-month follow-up, she experienced no new thrombotic events and maintained clinical quiescence of both APS and SLE while continuing anticoagulation. Persistent low-level CAR-T cell presence (0.53% of CD3^+^ T cells) and sustained B cell aplasia (<1 CD19^+^ B cell/μL) suggest durable immunologic reprogramming.

Furthermore, a 67-year-old woman with a 29-year history of APS, marked by recurrent thromboembolic events despite therapy with warfarin, was diagnosed with relapsed/refractory DLBCL [42]. After the failure of multiple chemotherapy regimens, CD19-directed CAR-T cell therapy (axicabtagene ciloleucel) was administered. Post-infusion, she developed only grade 1 CRS without thrombosis and achieved the complete remission of lymphoma by day 30. Remarkably, the elevated levels of anticardiolipin IgM antibodies normalized following CAR-T therapy, and she discontinued anticoagulation without further thrombotic events. At 12-month follow-up, both her APS serologies and lymphoma remained in remission. Flow cytometry confirmed ongoing CD19^+^ B cell aplasia, supporting a sustained immunologic reset. The above-described cases highlight the potential of CAR-T cell therapy to simultaneously treat refractory B cell malignancy and autoimmune disorders like APS through targeted B cell depletion.

### 3.6. Primary Sjögren’s Syndrome (pSS)

pSS is characterized by B cell hyperactivity, the production of anti-Ro/SSA and anti-La/SSB autoantibodies, and the progressive lymphocytic infiltration of exocrine glands leading to the development of sicca symptoms [43]. Sheng et al. reported a case presenting a 76-year-old female with pSS and relapsed DLBCL treated with CD19-directed CAR-T cells (axi-cabtagene ciloleucel) following fludarabine/CYC LDC and bridging radiotherapy [44]. Before treatment, the disease activity index (ESSDAI) was 5. During the early-post-infusion period, grade 2 CRS (fever, hypotension, cardiac decompensation), managed with three doses of tocilizumab, was developed, while grade 1 ICANS (tremor, dysgraphia) was controlled with corticosteroids and levetiracetam. Hematologic toxicities (anemia, thrombocytopenia) resolved within six weeks, and no infectious events were described. By day 28, the PET-CT scan confirmed the achievement of complete DLBCL metabolic remission. Concurrently, pSS features improved: sicca symptoms abated by one month, anti-Ro52 autoantibodies were negative by day 90, and serum cytokine levels normalized by month 1. Moreover, by month 3, the ESSDAI score decreased from 5 to 2, and the patient achieved a drug-free state for pSS. These findings underscore the potential of CD19 CAR-T cell therapy not only to treat concurrent B cell malignancies but also to reverse autoimmune pathology in pSS through targeted B cell depletion and immunomodulation.

### 3.7. Juvenile Idiopathic Arthritis (JIA) and Juvenile Dermatomyositis (JDM)

Pediatric rheumatic diseases constitute a unique challenge for CAR-T cell immunotherapy, given the lower immune reserves compared to adult individuals and the ongoing development of the immune system [45,46]. A refractory case of anti-melanoma differentiation-associated protein 5-positive dermatomyositis (MDA5^+^ DM), complicated by rapidly progressive ILD in a 12-year-old female, was managed with the infusion of second-generation anti-CD19 CAR-T cells (ARI-0001) following stabilization of the patient with high-dose corticosteroids, tacrolimus, and intravenous immunoglobulin [47]. Despite complete peripheral B cell aplasia at the time of infusion, in vivo CAR-T expansion was detected by flow cytometry, indicating the effective targeting of residual CD19^+^ B cells within inflamed tissues. Notably, significant CRS or ICANS were not occurred. Additionally, over an 11-month follow-up period, the patient exhibited remarkable improvement in cutaneous manifestations, muscle strength, respiratory function, and neurologic symptoms, all while remaining off immunosuppressive medications. This case suggests that anti-CD19 CAR-T therapy might achieve durable, drug-free remission in aggressive pediatric MDA5^+^ dermatomyositis-associated RP-ILD. In Table 1, an overview of the published case reports/series concerning CAR-T cell therapy for autoimmune rheumatic disorders is presented. 

## 4. CAR-T Cell Products in Autoimmune Rheumatic Disorders: Developments and Ongoing Clinical Trials

Several clinical trials are being conducted for the evaluation of CAR-T cell therapy in autoimmune rheumatic disorders, concerning both adult and pediatric populations. As mentioned above, CAR-T cells targeting several antigens expressed on the B cell surface might have a substantial role in the treatment of several rheumatic disorders, given the important role of these molecules in the pathogenesis of the aforementioned diseases.

### 4.1. Anti-CD19 CAR-T Cell Products

CD19 is a glycoprotein that has a substantial role in the enhancement of signaling via the B cell receptor (BCR) and, thus, affects several functions of B cells, including their differentiation, secretion, and production of antibodies and memory development [48]. Additionally, CD19 is associated with CD21 and CD81, forming a multimolecular signaling complex, while its expression varies on the B cell’s subtype and activation status [49]. It has been shown that CD19 is highly expressed on mature and memory B cells, as well as plasmablast cells producing antibodies, and is also identified on specific pre-B and plasma cells, mainly those lacking the expression of the CD20 molecule [50]. CD19 as an activator of phosphoinositide 3-kinase (PI3K) acts as a signaling-activating molecule, essential for the proliferation and survival of normal, autoreactive, and neoplastic B cells [51].

Beyond hematological malignancies, the expression of CD19 has been found to be increased in several autoimmune rheumatic disorders. In systemic sclerosis (SSc), CD19 expression levels have been correlated with SSc severity, fibrosis, and autoantibody levels [52]. Furthermore, various genetic variants of CD19 have been associated with susceptibility to SSc development [53]. In different experimental models, it has been shown that the enhancement of CD19 signaling results in the amplification of B cell activation, contributing to the fibrosis of the skin and lungs, while CD19 deficiency has been found to lead to the normalization of these pathological features [54]. At the same time, the upregulation of CD19 might induce local inflammatory responses through C-X-C motif chemokine receptor 3 (CXCR3) expression in autoreactive B cells at the affected tissues [55]. Thus, CD19 therapeutic inhibition might have a crucial role in SSc management. Inebilizumab is a monoclonal antibody that can bind to CD19 and deplete CD19-expressing B cells, and has been shown as an effective and therapeutic option for the management of patients with neuromyelitis optica spectrum disorder [56,57]. Inebilizumab’s role in the treatment of SSc is assessed in several ongoing clinical trials, aiming to evaluate its safety and efficacy in SSc, especially in those with severe skin and lung involvement [52].

B cells also play a critical role in SLE pathogenesis; thus, antigens expressed on the B cell surface have been used as therapeutic targets in this clinical entity [58]. The involvement of CD19 in B cell activation, maturation, and signaling, as described above, highlights the potential of its usage as a therapeutic target in this field. Based on this rationale, Obexelimab, a monoclonal antibody targeting CD19 and leading to the inhibition of B cell activation, was assessed in a phase 2 randomized, placebo-controlled trial in patients with moderate-to-severe SLE [59,60]. Nevertheless, despite that the treatment was generally well tolerated, the primary endpoint, i.e., maintenance of disease activity improvement, was not achieved [60]. Resistance against monoclonal antibodies might be attributed to the incomplete depletion of B cells in secondary lymphoid organs [61]. CD19-directed CAR-T cell products have been found capable of eliminating B cells in both peripheral blood and lymphoid tissues, offering a potential strategy to overcome this limitation [62].

The expression of CD19 antigen in various stages of B cell maturation, including its expression in plasmablasts, the experimental findings regarding CD19 therapeutic inhibition in some autoimmune rheumatic disorders, and the ability of CAR-T cells to eliminate autoreactive B cells in secondary lymphoid organs have led to the development of several anti-CD19 CAR-T cell products in this field. As described above, clinical data concerning the safety and efficacy of these CAR-T products in patients with rheumatic disorders are encouraging, while several phase I/II clinical trials are ongoing in several clinical entities (Supplementary Materials, Table S1).

The safety and efficacy of anti-CD19 CAR-T cell products are being examined in various rheumatic disorders, including SLE, SLE-LN, SSc, IIM, pSS, AAV, RA, and APS. Nevertheless, in some of the ongoing clinical trials, anti-CD19 CAR-T cell products are also being examined for patients with other autoimmune diseases, such as immune thrombocytopenia, primary biliary cholangitis, IgG4-related disease, neuromyelitis optica, multiple sclerosis, and myasthenia gravis, given the common pathophysiological mechanism between these clinical entities. In the majority of the ongoing studies, adult patients are being enrolled, while in some of them, the safety of anti-CD19 products in pediatric patients is being investigated [63,64]. Moreover, another interesting point investigated is the comparison of outcomes between patients who received LDC before CAR-T cell infusion versus those who did not, examining also the possible impact of LDC on disease control rates [65,66]. Tocilizumab prophylaxis prior to anti-CD19 infusion is also examined in a phase I clinical trial [67]. Additionally, while most of the ongoing clinical trials have a single-arm patient group, in NCT06475495 (phase I/II), an anti-CD19 CAR-T cell product is being compared with rituximab [68].

### 4.2. Anti-BCMA CAR-T Cell Products

The soluble form of B cell-activating factor (BAFF) has a pivotal role in B cell activation and survival by binding to cell surface receptors, including B cell maturation antigen (BCMA) [69]. Increased levels of BAFF lead to the persistence of autoreactive B cells and the generation of plasmablasts and plasma cells, which are implicated in the pathogenesis of several autoimmune rheumatic disorders, including SLE [70]. Moreover, BCMA is primarily expressed in plasmablasts, plasma cells, and some memory B cells, and upon activation, it leads to the upregulation of B cell activation [71]. Specifically, BCMA activation triggers various signaling pathways and mainly nuclear factor kappa-light-chain-enhancer of activated B cells (NF-κB), supporting cell proliferation and survival [72]. Interestingly, in SLE, levels of soluble BCMA (sBCMA) have not only been found to be higher in patients versus healthy controls but also have been associated with disease severity [73,74,75]. Additionally, in the study of Sanges et al., levels of sBCMA have been reported to be elevated in SSc patients with pulmonary arterial hypertension compared to the rest of the study population (*p* = 0.03) [76].

Based on the pathogenic role of BCMA via the activation of B cells in several autoimmune rheumatic disorders and the success of anti-BCMA CAR-T products in MM, various clinical trials are ongoing examining the safety and efficacy of these products in this field (Supplementary Materials, Table S2) [77].

### 4.3. Anti-CD20, CD22, and CD70 CAR-T Cell Products

The CD20 is a protein expressed on B cells in several stages of their maturation process, from the pre-B cell stage to mature memory B cells stage, playing a key role in the activation, proliferation, and differentiation of B cells [78]. As mentioned above, in rheumatic disorders such as RA and SLE, B cell activation contributes to disease pathogenesis through various processes, including autoantibody production, antigen presentation, cytokine release, and T cell activation [79]. Anti-CD20 monoclonal antibodies, such as rituximab, target and deplete B cells expressing CD20, i.e., not plasma cells and early B cell precursors, allowing for the maintenance of long-term humoral immunity and B cell regeneration post-treatment. Rituximab’s safety and real-world efficacy have been shown in several autoimmune rheumatic disorders [80,81]. However, rituximab’s chimeric structure can lead to the development of human anti-chimeric antibodies, potentially reducing their efficacy and tolerability due to increased immunogenicity [82]. Thus, CAR-T cell products targeting CD20 might be useful for the management of these clinical conditions [83]. In ongoing clinical trials, bispecific (dual target) and trispecific (triple target) CAR-T cell products targeting CD20 along with other B cell markers, including CD19, CD22, and BCMA, are being investigated. Additionally, in a phase I clinical trial, ADI-001, an allogeneic anti-CD20 CAR-T product, is being examined for patients with various rheumatic disorders [84].

CD22 is a protein, expressed on the majority of mature B cells, functioning as a co-receptor of BCR [85]. CD22 has an important role in the regulation of B cell responses to antigens [86]. After the binding of an antigen, CD22 becomes phosphorylated and activates downstream signaling pathways involving phosphatases [87]. Furthermore, in experimental studies, it has been shown that a loss of CD22 leads to B cell hyperactivation, implicating dysfunction in the development of autoimmune diseases [85]. As a result, the modulation of CD22 activity might also be a therapeutic target for rheumatic disorders [83]. LCAR-AIO is a trispecific CAR-T cell targeting CD19, CD20, and CD22, and its safety is being examined in phase I clinical trials for R/R SLE and other autoimmune disorders [88,89].

In autoimmune rheumatic disorders, such as SLE, CD70 (a costimulatory molecule typically expressed on T cells upon their activation) is overexpressed on B cells, contributing to disease pathogenesis. CD70/CD27, a receptor binding to the CD70 signaling pathway, has a significant role in the regulation of immune system responses by promoting T cell survival, B cell activation, and the differentiation of B cells into plasma cells. The therapeutic blockage of CD70/CD27 could lead to the inhibition of memory B cell differentiation into plasma cells; thus, CD70 is a promising therapeutic target. A phase I clinical trial aims to evaluate the safety and effectiveness of BCMA/CD70 bispecific CAR-T cells in pediatric patients with R/R SLE [90]. Additionally, an anti-CD70 universal (allogeneic) CAR-T cell product is being investigated for R/R SLE in a phase I clinical trial (Table 2) [91]

### 4.4. Bispecific (Dual Target) and Trispecific (Triple Target) CAR-T Cell Products

**Figure 1:**
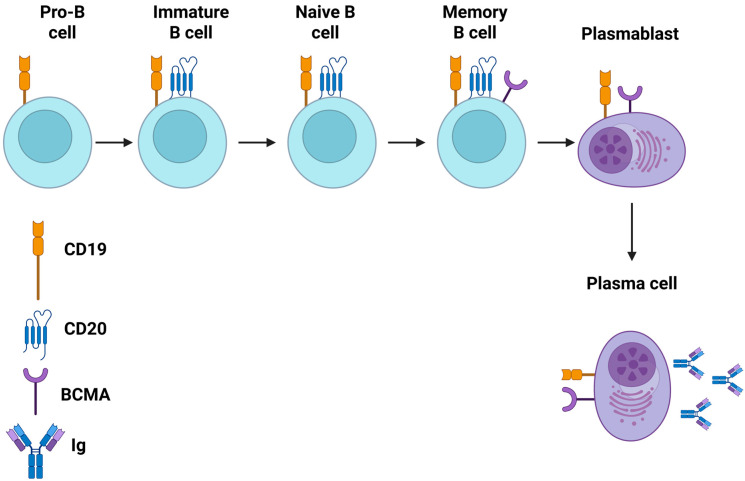
Mapping of the stages of B cell development—from early pro-B cells in the bone marrow through germinal center maturation to long-lived plasma cells—and highlighting of the surface antigens (e.g., CD19, CD20, BCMA) exploited by current CAR-T constructs. Moreover, the schematic illustrates how choice of antigen influences both the breadth of B-lineage deletion and the potential for on-target, off-tumor effects on normal B cell subsets. BCMA: B cell maturation antigen; CAR: chimeric antigen receptor T (created in BioRender. Evangelidis, P. (2025) https://BioRender.com/p17x35i).

**Figure 2:**
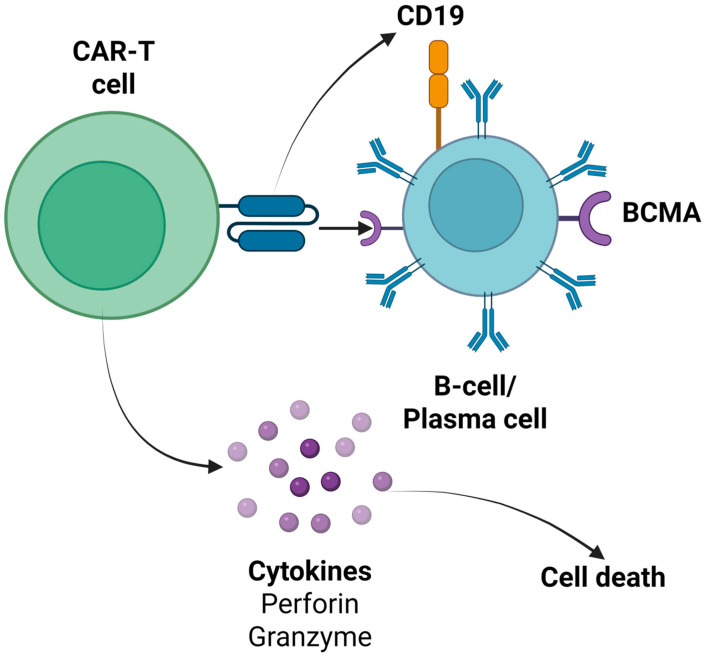
This diagram contrasts the activity of CD19-directed versus BCMA-directed CAR-T cells: anti-CD19 CAR-T targets CD19^+^ B cells at all developmental stages up to the plasma blast phase, while anti-BCMA CAR-T selectively targets BCMA^+^ plasma cells. It depicts CAR engagement, immunological synapse formation, and downstream cytotoxic pathways—perforin/granzyme release and death-receptor signaling—leading to the elimination of pathogenic B-lineage cells. BCMA: B cell maturation antigen; CAR: chimeric antigen receptor T (created in BioRender. Evangelidis, P. (2025) https://BioRender.com/86mtefl).

Bispecific and trispecific CAR-T cell products constitute the advanced generations of CAR-T immunotherapy, aiming to enhance their efficacy and overcome potential limitations such as antigen escape [92]. Bispecific CAR-T cells can recognize two distinct antigens simultaneously, while trispecific CAR-T cells target three different antigens, improving tumor recognition [93]. For this aim, tandem CARs (a single receptor recognizing multiple antigens) or co-expressed CARs (multiple distinct CARs on one T cell) can be used [94]. In the field of autoimmune rheumatic disorders, several bispecific and trispecific CAR-T cell products, targeting both pathogenic B cells and plasma cells by combining antigens, such as CD19, BCMA, CD20, CD22, and CD70, are being investigated in phase I/II clinical trials (Supplementary Material, Table S3). Additionally, it must be underlined that some ongoing studies are examining the role of these CAR-T cell products in neurological and renal autoimmune disorders, given the fact that the common pathogenetic mechanisms are implicated [91,95]. In Figure 1, the main CAR-T cell products’ target antigens of B cells, as expressed in various stages of the maturation process, are described. Moreover, in Figure 2, the mechanism of action of anti-CD19 and anti-BCMA CAR-T cell products is summarized [96,97,98,99,100,101,102,103,104,105,106,107,108,109,110,111,112,113,114,115,116].

## 5. Conclusions and Future Perspectives

In conclusion, the current body of evidence—comprising mainly case reports, small case series, and early-phase trials—suggests that CD19- and BCMA-directed CAR-T therapy can induce sustained remissions in patients with refractory B cell-driven autoimmune rheumatic diseases, although response rates appear to vary markedly between entities such as SLE, SSc, IIMs, APS, and pSS. A notable proportion of patients achieved complete clinical remission, normalization of pathogenic autoantibodies, and steroid- or immunosuppressant-free disease control, with generally mild-to-moderate CRS and ICANS managed effectively using tocilizumab, corticosteroids, or supportive care. Severe infections were uncommon and largely linked to transient neutropenia rather than persistent hypogammaglobulinemia, and the overall toxicity trends in the autoimmune cohort are lower than those seen in hematology, perhaps reflecting a lower antigen burden and attenuated inflammatory milieu. However, these promising findings stem from limited, heterogeneous studies without randomized controls, and variability in CAR constructs, lymphodepletion regimens, and follow-up durations complicate cross-study comparisons. Long-term safety—particularly regarding prolonged cytopenias and oncologic risks—remains uncertain.

Because CAR-T is generally reserved as a last-line option after multiple immunosuppressive regimens, careful patient selection—mirroring eligibility frameworks in hematologic malignancies—is essential. Candidates should have refractory or recalcitrant disease despite ≥2–3 prior lines of therapy, including at least one B cell-targeted agent, and must complete appropriate washout periods (e.g., ≥4 weeks for biologics, ≥2 weeks for small-molecule inhibitors) to minimize carryover immunosuppression. Baseline hematologic parameters should meet minimum thresholds (ANC ≥ 1000/µL; platelets ≥ 75,000/µL), and peripheral blood collections require sufficient T cell numbers (CD3^+^ T cells ≥ 150 cells/µL) and, ideally, detectable CD19^+^ B cells (e.g., ≥50 cells/µL) to ensure successful leukapheresis and manufacturing. In addition, patients must have acceptable end-organ function (e.g., LVEF ≥ 50%; creatinine clearance ≥ 30 mL/min; AST/ALT ≤ 2.5 × ULN) and no uncontrolled infections or active malignancies. Applying these criteria helps optimize CAR-T yield, balance efficacy against the risks of profound lymphodepletion, and improve overall safety in heavily pretreated autoimmune cohorts.

Despite their transformative potential, CAR-T therapies currently exhibit the least favorable safety and cost-effectiveness profiles among available treatments for autoimmune rheumatic diseases. The risk of severe acute toxicities—cytokine release syndrome, neurotoxicity, and prolonged cytopenias—remains higher than with conventional biologics (e.g., anti-TNF, anti-CD20) or autologous hematopoietic stem cell transplantation (auto-HCT), requiring inpatient monitoring, intensive supportive care, and specialized infrastructure. Moreover, the individualized manufacturing process—leukapheresis, viral transduction, ex vivo expansion, and quality testing—drives unit costs into the high six-figure range, substantially exceeding that of off-the-shelf monoclonal antibodies or even auto-HCT. While auto-HCT also entails cytopenia and infection risks, its one-time procedure and well-established logistics generally translate into lower overall expenditures and more predictable safety outcomes. Finally, long-term cost-effectiveness data for CAR-T in autoimmunity are lacking, underscoring the need for head-to-head trials and health economic analyses to determine whether the depth and durability of CAR-T-induced remissions justify its financial and safety trade-offs.

This review provides a comprehensive synthesis of CAR-T applications across diverse autoimmune rheumatic disorders—highlighting mechanistic rationales, real-world case outcomes, and an up-to-the-minute pipeline of ongoing trials [96,97,98,99,100,101,102,103,104,105,106,107,108,109,110,111,112,113,114,115,116]. However, important limitations should be acknowledged in the clinical application of CAR-T cell therapy in autoimmune rheumatic disorders. The available data are derived from small, often non-randomized cohorts and may be subject to publication bias favoring positive outcomes. Moreover, variability across studies—including differences in CAR design (e.g., CD28 vs. 4-1BB costimulatory domains), targeted antigens (CD19 vs. dual-target constructs such as CD19/BCMA), and LDC regimens—complicates direct comparisons and generalizability. Additionally, the long-term safety of CAR-T therapy in nonmalignant populations remains incompletely characterized, particularly concerning delayed neurotoxicity, prolonged cytopenias, and the potential risk of secondary malignancies.

Future research should prioritize randomized controlled trials comparing CAR-T therapy with conventional B cell-depleting biologic agents to better define its relative efficacy and safety. Moreover, other future research agendas might include the following:

- The role of different LDC regimens in CAR-T cell therapy outcomes and disease control.
- The potential role of tocilizumab prophylaxis in patients who receive CAR-T cell therapy for autoimmune rheumatic disorders.
- Direct comparison of CAR-T cell products with auto-HCT in patients with SSc, and potentially with other autoimmune diseases.
- The endothelial activation and stress index (EASIX) and its modified version (mEASIX) in the prediction of clinical outcomes of these patients, as has been successfully used in the setting of CAR-T cell therapy for hematological malignancies [117].

## Figures and Tables

**Table 1:** Overview of the published case reports/series concerning CAR-T cell therapy for autoimmune rheumatic disorders.

First Author (Year)	Study Design-CAR-T Cell Product	Patient Characteristics	Outcomes	CRS (Grade)	ICANS (Grade)	Infections During Follow-Up	Prolonged Hematological Toxicity
[23]	Single-case report; refractory SLE → autologous CD19-CAR (1.1 × 10^6^ cells/kg)	20-y-old female with refractory SLE (class IIIA lupus nephritis, nephrotic syndrome, pericarditis, pleuritis, malar rash, arthritis, and prior Libman–Sacks endocarditis). Failed hydroxychloroquine, steroids, CYC, MMF, tacrolimus, belimumab, and rituximab.	SLEDAI ↓ 18 → 0 by day 44; complete B cell depletion (≥44 days); anti–dsDNA normalized; complement (C3/C4) normalized; proteinuria improved.	None reported (no CRS)	None reported	None reported	None reported
[24]	Prospective case series (five patients); refractory SLE → autologous CD19-CAR (1 × 10^6^ cells/kg)	Adults (4 F/1 M; median 22 y; baseline SLEDAI 8–16) with refractory SLE despite multiple immunosuppressants (such as CYC, MMF, tacrolimus, belimumab, and rituximab).	5/5 achieved SLEDAI 0 by month 3; anti-dsDNA and ANA normalized; proteinuria resolved; drug-free remission maintained (median follow-up: 8 months); reconstituted B cells naïve phenotype.	3/5 had grade 1 (fever, hypotension); 2/5 received tocilizumab	None reported	No serious infections (≥12 months follow-up)	No prolonged cytopenias or hypogammaglobulinemia survived beyond 3 months
[26]	Single-case report; refractory SLE in pregnancy → autologous CD19-CAR (1 × 10^6^ cells/kg)	32-y-old female with refractory SLE (serositis, nephritis, cytopenias, hypocomplementemia, and suspected CNS involvement) diagnosed during pregnancy; off all immunosuppression except low-dose steroids before CAR-T.	Achieved LLDAS by month 3; proteinuria resolved; anti-dsDNA undetectable; B cell reappearance at ~month 2 without disease flare.	None reported	None reported	None reported	None reported
[27]	Single-case report; severe CNS SLE → autologous CD19-CAR (1 × 10^6^ cells/kg)	21-y-old male with transverse myelitis and vasculitis from CNS SLE (SLEDAI: 22), refractory to standard of care.	Achieved SLEDAI 0 by week 12; full neurologic recovery; MRI lesions resolved; anti-dsDNA and IFN-α undetectable.	None reported	None reported	None reported	None reported
[28]	Single-case report; refractory SLE + DLBCL → dual-target CD19/BCMA-CAR (5.3 × 10^6^ cells/kg)	41-y-old female with 20-y refractory SLE complicated by stage IV DLBCL; intolerant to R-CHOP.	CAR-T expansion to ~8% lymphocytes; sustained B cell aplasia to ~9 months; complement C3/C4 normalized; ANA titers undetectable by weeks 9–37; IgG/A/M ↓; PET-CT CR of DLBCL at 4 months; SLE in remission at 23 months post-CAR-T infusion.	Minimal (not specified; no severe CRS)	None reported	None reported	No prolonged hematologic toxicity reported
[29]	Two-case pediatric report; refractory pediatric SLE → low-dose CD19-CAR (1 × 10^5^ cells/kg)	Patient 1: cutaneous ulcers, arthritis, macrophage activation syndrome, and nephrotic-range proteinuria Patient 2: hypertension, pleuritis, hematuria, and class IV LN. Both off immunosuppression before leukapheresis.	Patient 1: SLEDAI-2K 12 → 0 by month 4; C3 normalized by day 28; anti-dsDNA undetectable; cutaneous lesions resolved; proteinuria resolved. Patient 2: SLEDAI-2K 12 → 4; proteinuria 28 → 13.6 mg/kg/day; C3/C4 normalized; pleuritis and hematuria resolved; LN activity improved on biopsy. Both off immunosuppression at 4–5 months.	2/2 grade 1 CRS (fever); managed supportively	1/2 transient grade 1 (mild encephalopathy)	Transient hypogammaglobulinemia managed with IVIG	No prolonged cytopenias beyond month 2
[32]	Single-case report; refractory dcSSc → autologous CD19-CAR (1 × 10^6^ cells/kg)	60-y-old male with dcSSc: skin, pulmonary, and cardiac fibrosis; anti-RNA Pol III +; prior MTX (3 mo) and MMF (23 mo) failed; P-AH/ILD/digital ulcers. Renal impairment → reduced lymphodepletion (Flu 12.5 mg/m^2^ × 3 d, CYC 500 mg/m^2^ × 1 d).	mRSS ↓ progressively; Joint inflammation resolved; FVC ↑; pulmonary hemodynamics improved; troponin T stabilized; anti-RNA Pol III undetectable by month 3.	1/1 grade 1 (fever)	None reported	None reported	No persistent cytopenias (resolved by month 2)
[33]	Prospective case series (six patients); severe diffuse SSc → autologous CD19-CAR (1 × 10^6^ cells/kg)	Adults (median age: 45 y; 4 F/2 M) with severe diffuse SSc: skin (mRSS ≥ 20), ILD (HRCT > 15% lung), P21+AH, and joint involvement; refractory to ≥1 DMARD (e.g., MMF, CYC, nintedanib).	At 6 months: median ACR-CRISS improvement probability 100%; mRSS ↓ median 8 points (~31%); ILD extent ↓ 4% on HRCT; FVC ↑ 195 mL at the last follow-up.	1/6 grade 0; 3/6 grade 1; 2/6 grade 2	0/6	1/6 hospitalized for influenza + bacterial superinfection	No prolonged cytopenias beyond month 2
[34]	Single-case report; anti-Scl-70 + diffuse SSc → autologous CD19-CAR (third gen) (5 × 10^6^ cells/kg)	38-y-old female with anti-Scl-70 + diffuse SSc; progressive nonspecific ILD; prior MMF and nintedanib.	mRSS ↓ steadily over 11 months; FVC and DLCO ↑; CT → regression of GGO/fibrosis; ^68 Ga-FAPI PET uptake ↓; anti-Scl-70, CRP, hs-troponin T normalized; Fcγ-R-activating immune complexes disappeared.	1/1 grade 1 (fever)	None reported	None reported	No persistent cytopenias
[25]	Prospective cohort (15 patients); refractory SLE (*n* = 8), IIMs (*n* = 3), SSc (*n* = 4) → autologous CD19-CAR (1 × 10^6^ cells/kg)	SLE (*n* = 8): refractory, high SLEDAI, multiorgan involvement. IIMs (*n* = 3): antisynthetase syndrome (*n* = 2), IMNM (*n* = 1); refractory to CYC, rituximab, tacrolimus. SSc (*n* = 4): severe diffuse, refractory to standard DMARDs.	SLE (8/8): SLEDAI ↓ to LLDAS/remission by month 3; complement and anti-dsDNA normalized. IIM (3/3): CK ↓ normalized; muscle strength ↑; ACR-EULAR major response. SSc (4/4): EUSTAR ↓; mRSS ↓; ILD ↓ 4–6%; FVC ↑; steroid/DMARD discontinuation. All remissions sustained 12–29 months.	11/15 (73%) grade 1–2 (fever, mild hypotension); 1/15 grade 1 ICANS	1/15 grade 1	Mostly mild (upper respiratory, CMV reactivation—all resolved)	Transient cytopenias (grade 3–4) in 100%; resolved by month 2; transient hypogammaglobulinemia (IVIG support)
[36]	Single-case report; refractory RA + DLBCL → bispecific CD20/CD19-CAR (zamtocabtagene)	73-y-old male with long-standing RA refractory to methotrexate and tocilizumab; developed DLBCL. RA meds discontinued pre-lymphodepletion.	Rheumatoid factor ↓ 1200 → 13 IU/mL by month 1; drug-free RA remission by week 4 (persisted 12 months); DLBCL CR by week 48.	1/1 grade 1 (fever)	None reported	No serious infections	No prolonged cytopenias beyond month 2 (neutropenia managed with G-CSF)
[47]	Single-case report; pediatric JDM (MDA5^+^) → autologous CD19-CAR (ARI-0001)	12-y-old female with MDA5^+^ dermatomyositis complicated by rapidly progressive ILD; refractory to high-dose steroids, tacrolimus, and IVIG. Complete peripheral B cell aplasia at infusion.	Over 11 months: cutaneous lesions resolved; muscle strength ↑; respiratory function ↑; neurologic symptoms improved; remained off immunosuppressives.	None reported	None reported	None reported	No prolonged cytopenias
[38]	Single-case report; refractory anti-Jo-1 antisynthetase syndrome → CD19-CAR (1 × 10^6^ cells/kg)	41-y-old male with severe anti-Jo-1 antisynthetase syndrome: CK > 9000 U/L, ILD, high anti-Jo-1 titers; refractory to steroids, rituximab, tacrolimus, IVIG, and CYC.	CK ↓ 13,600 → 102 U/L by day 180; anti-Jo-1 331 → 5 U/mL; muscle strength normalized; ILD resolved on CT; functional tests normalized; sustained remission at 6 months; B cells reappeared naïve.	1/1 grade 1 (fever, myalgia, CK spikes); managed with tocilizumab	None reported	Received monthly IVIG for hypogammaglobulinemia; no serious infections	Transient cytopenias; resolved by month 2
[39]	Single-case report; anti-Jo-1 syndrome → CD19-CAR (1.23 × 10^6^/kg) + mycophenolate	41-y-old male with refractory anti-Jo-1 antisynthetase syndrome (7 y): severe ILD, muscle weakness and high anti–Jo-1; prior rituximab and AZA failed. MMF started on day 35 to co-target CD8^+^ T cells.	Muscle enzymes and inflammatory cytokines normalized by week 4; anti-Jo-1 ↓; IgA/IgG/IgM partially recovered; muscle strength and pulmonary function improved (MRI and PFTs); remission persisted at 8 months; steroid/DMARD discontinued.	None reported	None reported	None reported	No prolonged cytopenias beyond month 2
[40]	Phase I trial (first subject); anti-SRP IMNM → fully human CD19-CAR (CABA-201) (1 × 10^6^ cells/kg)	39-y-old male with refractory anti-SRP-positive IMNM: CK elevated, anti-SRP, anti-Ro-52; refractory to steroids, rituximab, tacrolimus, IVIG, and CYC.	CK normalized by week 8; muscle strength and endurance improved; anti-SRP-9/-54/-72 and anti-Ro-52 titers ↓; vaccine/pathogen Ab titers unchanged; B cells depleted and reappeared naïve.	None reported	None reported	None reported	No prolonged cytopenias
[41]	Single-case report; APS + SLE + relapsed aggressive B cell lymphoma → CD19-CAR (axi-cel)	65-y-old female with SLE (longstanding) and triple-positive APS (recurrent DVT) complicated by relapsed B cell lymphoma. On warfarin throughout.	At day 79: anticardiolipin IgG/IgM and anti-β2-GPI IgG/IgM undetectable; lupus anticoagulant and ANA negative; sustained B cell aplasia (<1 cell/μL); no new thromboses; lymphoma remission at 12 months; continued anticoagulation.	1/1 grade 1 (fever, hypotension); tocilizumab + dexamethasone	1/1 grade 4 (severe encephalopathy); resolved with high-dose steroids	Transient neutropenia; no serious infections	None beyond month 2
[42]	Single-case report; 29-y APS + relapsed DLBCL → CD19-CAR (axi-cel)	67-y-old female with 29-y APS (recurrent thromboses on warfarin); relapsed/refractory DLBCL after multiple chemo regimens.	DLBCL CR by day 30; anticardiolipin IgM normalized; warfarin discontinued; no thrombotic events to 12 months; sustained B cell aplasia; APS serology remained negative at 12 months.	1/1 grade 1 (fever)	None reported	No infections reported	No prolonged cytopenias beyond month 2
[44]	Single-case report; pSS + relapsed DLBCL → CD19-CAR (axi-cel) (2 × 10^6^ cells/kg)	76-y-old female with 10-y active primary Sjögren’s (ANA +/anti-Ro-52 +; ESSDAI 5) and relapsed DLBCL; prior R-CHOP, lenalidomide, and ICE + zanubrutinib.	DLBCL: CR by day 28 (Deauville 2); sustained to 6 months. pSS: anti-Ro-52 + → negative by day 90; cytokines (IL-6, IL-10, TNF-α, IFN-γ) normalized by month 1; ESSDAI 5 → 2 by month 3; xerostomia/xerophthalmia improved; drug-free at 3 months.	1/1 grade 2 (fever, hypotension, heart failure on day 6; tocilizumab × 3)	1/1 grade 1 (tremor, dysgraphia; steroids + levetiracetam)	None serious; transient cytopenias resolved by week 6; no infections reported (except one pneumonia in MG-2).	None beyond week 6

↓: decrease; ↑: increase; ACR-CRISS: American College of Rheumatology Composite Response Index in diffuse cutaneous systemic sclerosis; anti-β2-GPI: anti-beta-2 glycoprotein I antibody; anti-Scl-70: anti-topoisomerase I antibody; anti-dsDNA: anti-double-stranded DNA antibody; anti-SRP-9: anti-signal recognition particle 9 antibody; APS: antiphospholipid syndrome; BCMA: B cell maturation antigen; CAR: chimeric antigen receptor; CK: creatine kinase; CMV: cytomegalovirus; CNS: central nervous system; CR: complete response; CRP: C-reactive protein; CRS: cytokine release syndrome; CT: computed tomography; CYC: cyclophosphamide; dcSSc: diffuse cutaneous systemic sclerosis; DLBCL: diffuse large B cell lymphoma; DLCO: diffusing capacity of the lung for carbon monoxide; DMARD: disease-modifying antirheumatic drug; DVT: deep vein thrombosis; ESSDAI: EULAR Sjögren’s syndrome disease activity index; F: female; Flu: fludabarine; FVC: forced vital capacity; G-CSF: granulocyte colony-stimulating factor; GGO: ground-glass opacity; HRCT: high-resolution computed tomography; ICE: ifosfamide, carboplatin, and etoposide chemotherapy regimen; ICANS: immune effector cell-associated neurotoxicity syndrome; IFN-α: interferon-alpha; IFN-γ: interferon-gamma; IIMs: idiopathic inflammatory myopathies; IL-10: interleukin 10; IL-6: interleukin 6; IMNM: immune-mediated necrotizing myopathy; IVIG: intravenous immunoglobulin; JDM: juvenile dermatomyositis; LN: lupus nephritis; LLDAS: lupus low disease activity state; M: male; MDA5^+^: positive for melanoma differentiation-associated protein 5; MMF: mycophenolate mofetil; MRI: magnetic resonance imaging; mRSS: modified Rodnan skin score; MTX: methotrexate; PET-CT: positron emission tomography–computed tomography; pSS: primary Sjögren’s syndrome; RA: rheumatoid arthritis; R-CHOP: rituximab, cyclophosphamide, doxorubicin, vincristine, and prednisone chemotherapy regimen; SLE: systemic lupus erythematosus; SLEDAI: systemic lupus erythematosus disease activity index; SSc: systemic sclerosis; TNF-α: tumor necrosis factor-alpha.

**Table 2:** A summary of the ongoing clinical trials on the role of anti-CD20 and CD70 CAR-T cell products in autoimmune rheumatic disorders.

Clinical Trial Registration Number, Reference	Country	Design Phase	CAR-T Cell Product	Autoimmune Rheumatic Disease	Primary Study Endpoints	Status
NCT06375993 [84]	United States	Open label, multiple-arm, phase I	ADI-001 (allogeneic anti-CD20)	- SLE-LN - SLE - SSc - AAV - IIMs - pSS	- Incidence and type of TEASs - Incidence of DLTs	Recruiting
NCT06946485 [91]	China	Open label, single-arm, early phase I	Allogeneic universal CHT101 (anti-CD70)	Relapsed/refractory SLE	- Incidence of TEAEs - Safety evaluation	Not yet recruiting

AAV: ANCA-associated vasculitis; ANCA: anti-neutrophil cytoplasmic antibodies; CAR-T: chimeric antigen receptor T cell therapy; DLTs: dose-limiting toxicities; IIMs: idiopathic inflammatory myopathies; pSS: primary Sjögren’s syndrome; SLE: systemic lupus erythematosus; SLE-LN: systemic lupus erythematosus–lupus nephritis; SSc: systemic sclerosis; TEAEs: treatment-emergent adverse events.

## Data Availability

No new data were created or analyzed in this study.

## Supplementary Materials

The following supporting information can be downloaded at: https://www.mdpi.com/article/10.3390/cells14161242/s1.
