# Supplementary material for: Chimeric Antigen Receptor T Cell Immunotherapy for Autoimmune Rheumatic Disorders: Where Are We Now?
Source: Cells. 2025 Aug 12;14(16):1242. doi: 10.3390/cells14161242 (PMC12384554; doi:10.3390/cells14161242)
Supplement: Supplementary file 1 [file cells-14-01242-s001.zip › cells-3781980_TableS1.pdf]

Table S1. A summary of the ongoing clinical trials on the role of anti-CD19 CAR-T cell products in autoimmune rheumatic disorders.

Clinical trial registration number, Reference	Country	Design-Phase	CAR-T cell product	Autoimmune rheumatic disease	Primary study endpoints	Status
NCT06585514 [1]	China	Open-label, single-arm, phase I/II	Anti-CD19	Refractory SLE	-Phase I: Type and incidence of DLT within 28 days after infusion<br>-Phase II: ORR	Recruiting
NCT06685042 [2]	Italy	Open-label, single-arm, phase I	Anti-CD19	-SLE<br>-SSc<br>-DM/PM	-CRS, ICANS incidence<br>-Incidence of infections, leukopenia, hypogammaglobulinemia<br>-ORR	Recruiting
NCT06691152 [3]	China	Open label, single-arm, phase I	Anti-CD19	Refractory SLE (pediatric patients)	Incidence, severity, and type of TEAEs	Recruiting
NCT06710717 [4]	Malaysia	Open label, single-arm, phase I	Anti-CD19	Severe refractory SLE	-Incidence of TEAEs<br>-Clinical remission	Not yet recruiting
NCT06711146 [5]	China	Open label, single-arm, early phase I	Metabolically Armed anti-CD19 (Meta10-19)	Severe active SLE	- MTD<br>-DCR	Recruiting
NCT06752876 [6]	United States	Open label, single-arm, phase I	CRISPR-edited allogeneic Anti-CD19 (CB-010)	Refractory SLE	Incidence and type of TEAEs	Recruiting
NCT06826430 [7]	China	Open label, single-arm, phase I	Inaticabtagene autoleucel (anti-CD19)	SLE related ITP	-Incidence and type of TEAEs<br>-RD for phase II trial	Not yet recruiting
NCT06839976 [8]	United States	Open label, single-arm, phase I-II	Anti-CD19	-Refractory SLE<br>-Refractory SLE-LN (pediatric patients)	Incidence of DLT	Recruiting
NCT06886919 [9]	China	Open label,	IC19 (Anti-CD19)	Refractory SLE	-Incidence of TEAEs	Not yet recruiting

		single-arm, early phase I			-DLT	
NCT06892145 [10]	China	Open label, single-arm, phase I	MC-1-50 (Anti-CD19)	Refractory SLE	-Incidence of TEAEs<br>-DLT	Not yet recruiting
NCT06904729 [11]	China	Open label, single-arm, phase III	Anti-CD19	Refractory/ recurrent SLE-LN	Incidence of TEAEs	Recruiting
NCT06913608 [12]	United States	Open label, randomized, phase I	CLBR001+SWI019 (anti-CD19)	-SLE<br>-SSc<br>-IIM	Incidence of TEAEs	Not yet recruiting
NCT06581198 [13]	Multiple countries	Open label, randomize, phase II	Rapcabtagene Autoleucel (anti-CD19)	Active, refractory SLE-LN	Clinical response at week 52	Recruiting
NCT06549296 [14]	China	Open label, single-arm, phase I	RD06-04 (anti-CD19)	Active<br>-SLE<br>-SSc<br>-AAV<br>-IM<br>-pSS	Incidence of TEASs	Recruiting
NCT06544330 [15]	United States	Open label, single-arm, phase I	SYNCAR-001 + STK-009 (anti-CD19 co-expressing an engineered IL-2 beta receptor and an engineered pegylated IL-2)	-SLE<br>-SSc	- Incidence of TEASs<br>-DLT	Recruiting
NCT06513429 [16]	China	Open label, single-arm, phase not applicable	IM19 (anti-CD19)	Refractory SLE	-Incidence of TEASs<br>- Change from baseline of SLEDAI-2K score	Recruiting
NCT06465147 [17]	United States	Open label, single-arm, phase I	SCRI-CAR19v3 (anti-CD19)	Refractory SLE (pediatric patients)	-Incidence of TEASs<br>-Rate of SCRI-CAR19v3 manufacturing success	Recruiting
NCT06420154 [18]	China	Open label, single-arm, early phase I	Anti-CD19	Relapsed/ refractory<br>-SLE<br>-pSS<br>-Diffuse SSc	-Incidence of TEASs<br>-DLT	Not yet recruiting

				-IIM		
NCT06417398 [19]	China	Open label, single-arm, early phase I	UTAA09 (anti-CD19)	Relapsed/ refractory -SLE -IIM -SSc -pSS -RA -ITP -PBC	-Incidence and type of TEASs	Not yet recruiting
NCT06373991 [20]	China	Open label, single-arm, phase I	ATHENA/ ET-901 (allogeneic anti-CD19)	SLE	-Incidence and type of TEASs -DLT	Not yet recruiting
NCT06342960 [21]	Germany	Open label, single-arm, phase I/ II	KYV-101 (anti-CD19)	Refractory SLE-LN	-Incidence and type of TEASs - Incidence of DLTs	Recruiting
NCT06340490 [22]	China	Open label, single-arm, phase I	RJMty19 (allogeneic anti-CD19)	Refractory SLE	-Incidence and type of TEASs -DLT -MTD	Not yet recruiting
NCT06333483 [23]	Spain, United Kingdom	Open label, single-arm, phase I	Obecabtagene autoleucel (anti-CD19)	Severe/ refractory SLE	-Incidence and type of TEASs -DLT	Recruiting
NCT06316791 [24]	China	Open label, single-arm, early phase I	CNCT19 (anti-CD19)	Refractory -SLE (ITP, SLE-LN) -DM -AAV	-Incidence and type of TEASs	Recruiting
NCT06310811 [25]	China	Open label, single-arm, phase not applicable	RD06-04 (anti-CD19)	Severe active SLE	-Incidence and type of TEASs -DLT	Recruiting
NCT06308978 [26]	United States	Open label, multiple arm, phase I	FT819 (allogeneic anti-CD19)	Severe active -SLE -AAV -IIM -SSc	-Incidence and type of TEASs - Incidence of DLTs -Phase II RD	Recruiting
NCT06297408 [27]	China	Open label, single arm, phase I	Anti-CD19	Severe active SLE	- Incidence of DLTs -Phase II RD	Not yet recruiting
NCT06294236 [28]	United States	Open label, single arm, phase I	SC291 (allogeneic anti-CD19)	Severe -SLE -SLE-LN -AAV	- Incidence and type of TEASs - Incidence of DLTs	Recruiting

NCT06222853 [29]	China	Open label, single arm, phase I	Anti-CD19	SLE	Incidence and type of TEASs	Recruiting
NCT06189157 [30]	Germany	Open label, single arm, phase I/ IIa	MB-CART19.1 (Anti-CD19)	Refractory SLE	Phase I:<br>- Incidence of DLTs<br>-Phase II RD<br>Phase II:<br>Remission rates	Recruiting
NCT06150651 [31]	Thailand	Open label, single arm , phase I	PiggyBac (anti-CD19)	Refractory SLE	Incidence and type of TEASs	Recruiting
NCT06121297 [32]	-United States<br>-Spain	Open label, single arm , phase I/ II	CABA-201 (anti-CD19)	Active<br>-SLE<br>-SLE-LN	Incidence of TEASs	Recruiting
NCT06106906 [33]	China	Open label, single arm, phase I/ II	Anti-CD19	Active SLE	Incidence and type of TEASs	Recruiting
NCT06106893 [34]	China	Open label, single arm , phase I/ II	Anti-CD19 universal CAR-γδT	Active SLE	Incidence and type of TEASs	Recruiting
NCT05988216 [35]	China	Open label, single arm , phase not applicable	BRL-301(universal anti-CD19)	Refractory SLE	Incidence and type of TEASs	Recruiting
NCT05938725 [36]	United States	Open label, single arm, phase I/ II	KYV-101 (anti-CD19)	Refractory SLE-LN	Phase I:<br>- Incidence of DLTs<br>- Incidence and type of TEASs<br>Phase II:<br>assessment of efficacy	Recruiting
NCT05930314 [37]	China	Open label, single arm, Early phase I	CNCT19 (anti-CD19)	Refractory SLE	Incidence and type of TEASs	Enrolling by invitation
NCT05869955 [38]	-United States<br>-Belgium<br>-France<br>-Germany<br>-Italy<br>-Spain	Open label, single arm, phase I	CC-97540 (anti-CD19)	Severe, refractory:<br>-SLE<br>-IIM<br>-SSc	- Incidence of DLTs<br>- Incidence and type of TEASs<br>-Recommended phase II dose	Recruiting
NCT05859997 [39]	China	Open label, single arm,	BRL-301 (universal anti-CD19)	Refractory:<br>-SLE<br>- pSS	- Incidence of DLTs	Enrolling by invitation

		phase not applicable		-SSc<br>-IIM<br>-AAV<br>-APS	- Incidence and type of TEASs	
NCT06828042 [40]	China	Open label, single arm, phase I/ II	Anti-CD19	-SLE<br>-SSc<br>-pSS<br>-AAV<br>-IIM<br>-APS	- Incidence of DLTs<br>- Incidence and type of TEASs	Not yet recruiting
NCT06821659 [41]	China	Open label, single arm, phase I/ II	UWD-CD19 (universal anti-CD19)	-SLE<br>-SSc<br>-IIM<br>-AAV<br>-pSS	- Incidence of DLTs<br>- Incidence and type of TEASs	Not yet recruiting
NCT06475495 [42]	Germany	Open label, randomized, controlled, phase I/II	Anti-CD19 versus Rituximab	RA	Incidence and type of TEASs	Not yet recruiting
NCT06361745 [43]	China	Open label, single arm, phase not applicable	UTAA09 (allogeneic anti-CD19 CAR-γδ T)	-SLE<br>-IIM<br>-SSc<br>-pSS<br>-IgG4-related disease	Incidence and type of TEASs	Recruiting
NCT06056921 [44]	China	Open label, single arm, Phase I	Anti-CD19	-SLE<br>-pSS<br>-SSc<br>-DM<br>-AAV	- Incidence of DLTs<br>- Incidence and type of TEASs<br>-Clinical response rates	Recruiting
NCT06414135 [45]	China	Open label, single arm, phase I	Relmacabtagene Autoleucel (anti-CD19)	Refractory-progressive SSc	- Incidence of DLTs<br>- Incidence and type of TEASs	Recruiting
NCT06400303 [46]	United States	Open label, single arm, phase I/ II	KYSA-5 (anti-CD19)	SSc	Phase I:<br>- Incidence of DLTs<br>- Incidence and type of TEASs<br>Phase II: Clinical efficacy	Recruiting
NCT06328777 [47]	United States	Open label, single arm, phase I/ II	CABA-201 (anti-CD19)	Active SSc	Incidence and type of TEASs	Recruiting
NCT06792344 [48]	-China	Open label, single arm,	Anti-CD19	Refractory SSc	Clinical efficacy	Recruiting

	- Christmas Island	phase I				
NCT06548620 [49]	China	Open label, single arm, early phase I	RD06-04 (anti-CD19)	-SLE<br>-SSc<br>-AAV<br>-IIM<br>-pSS<br>-NMO<br>-RRMS<br>- Myasthenia Gravis	Incidence and type of TEASs	Not yet recruiting
NCT06686524 [50]	China	Open label, single arm, phase I	Anti-CD19	Refractory juvenile DM	- Incidence of DLTs<br>- Incidence and type of TEASs<br>- Clinical efficacy	Recruiting
NCT06569472 [51]	China	Open label, single arm, phase I	Anti-CD19	Juvenile DM	- Incidence and type of TEASs<br>-Clinical efficacy<br>-Duration of clinical response	Recruiting
NCT06298019 [52]	United States	Open label, single arm, phase I	KYV-101 (anti-CD19)	Adult DM	Incidence of TEASs	Not yet recruiting
NCT06154252 [53]	-United States<br>-United Kingdom<br>-	Open label, single arm, phase I/ II	CABA-201 (anti-CD19)	IIM (adult and juvenile)	Incidence and severity of TEASs	Recruiting
NCT06868290 [54]	-United States<br>-Israel<br>-Japan<br>-Singapore	Open label, randomized, phase II	Anti-CD19 versus other therapeutic agents	Severe/ active IIM	EFS	Recruiting
NCT06590545 [55]	Germany	Open label, single-arm, phase I/ II	KYV-101 (Anti-CD19)	Active/ treatment-refractory AAV	-Incidence and severity of TEASs<br>- ANCA seroconversion rate	Not yet recruiting
NCT06508346 [56]	China	Observational -patient Registry]	Anti-CD19	Refractory AAV	Incidence and severity of TEASs	Recruiting
NCT06569472 [51]	China	Open label, single arm, phase I	Anti-CD19	Juvenile DM	- Incidence and type of TEASs	Recruiting

					-Clinical efficacy<br>-Duration of clinical response	
NCT06347718 [57]	Germany	Open label, single-arm, phase I/ II	Anti-CD19	-SLE<br>-SSc<br>-IIM	Incidence and type of TEASs	Recruiting
NCT06665256 [58]	-United States<br>-Italy<br>-Israel<br>-Germany<br>-Japan<br>-Singapore<br>-Japan	Open label, randomized, phase II	Rapcabtagene autoleucel (anti-CD19) versus standard of care	Severe refractory IIM	Clinical efficacy	Recruiting

AAV: ANCA-associated vasculitis, ANCA: anti-neutrophil cytoplasmic antibodies, APS: antiphospholipid syndrome, CAR-T: chimeric antigen receptor T-cell therapy, CRISPR: clustered regularly interspaced short palindromic repeats, CRS: cytokine release syndrome, DCR: disease control rate, DLT: dose-limiting toxicity, DM: dermatomyositis, EFS: event-free survival, ICANS: immune effector cell–associated neurotoxicity syndrome, IIM: idiopathic inflammatory myopathies, ITP: immune thrombocytopenia, MTD: maximum tolerated dose, NMO: neuromyelitis optica, ORR: objective response rate, PBC: primary biliary cholangitis, PM: polymyositis, pSS: primary Sjogren's syndrome, RA: rheumatoid arthritis, RD: recommended dose, RRMS: relapsing-remitting multiple sclerosis, SLE: systemic lupus erythematosus, SLEDAI-2K: systemic lupus erythematosus disease activity index 2000, SLE-LN: systemic lupus erythematosus–lupus nephritis, SSc: systemic sclerosis, TEAES: treatment-emergent adverse events.

NCT06694298 [59]		-Type and incidence of DLT<br>-Incidence of TEAEs<br>-RD
NCT06902844 [60]	Open label,	Response rate 4-6 months post treatment

NCT0649738 7 [61]	Open label, single-arm, phase I	-Refractory SLE-LN	-Incidence of TEASs -Safe CAR-T infusion dose
NCT0634075 0 [62]	Open label, single-arm, phase I	SLE	-Incidence and type of TEASs -Phase II RD

NCT06277427 [63]	Open label, multiple arm, phase I	-SLE-LN<br>-AAV	- Incidence and type of TEASs<br>- Incidence of DLTs
NCT06038474 [64]	Open label, single arm, phase II	SLE	Incidence and type of TEASs

AAV: ANCA-associated vasculitis, ANCA: anti-neutrophil cytoplasmic antibodies, BCMA: B Cell maturation antigen, CAR-T: chimeric antigen receptor T-cell therapy, DLT: dose-limiting toxicity, RD: rheumatic disease, SLE: systemic lupus erythematosus, TEAEs: treatment-emergent adverse events.

References

1. Anti-CD19 Chimeric Antigen Receptor T Cells for Refractory Systemic Lupus Erythematosus.
2. Anti-CD19 CAR T-Cell Therapy in Refractory Systemic Autoimmune Diseases (CATARSIS).

3. Efficacy and Safety of CD19 UCAR T Cells in Refractory Systemic Lupus Erythematosus (SLE).
4. Autologous CD19 Car T-Cell Therapy For Severe Refractory Systemic Lupus Erythematosus (SLE).
5. Safety and Efficacy of Metabolically Armed CD19 CAR-T Cells (Meta10-19) in the Treatment of Moderate to Severe Active SLE Clinical Research.
6. CRISPR-Edited Allogeneic Anti-CD19 CAR-T Cell Therapy, in Patients With Refractory Systemic Lupus Erythematosus (GALLOP) (GALLOP).
7. Inaticabtagene Autoleucel Injection in the Treatment of Refractory Systemic Lupus Erythematosus-Related Immune Thrombocytopenia.
8. CD19-Directed Chimeric Antigen Receptor Autologous T Cells (CART19) for Lupus.
9. Clinical Study Evaluating the Safety and Efficacy of IC19 CAR-T Cell Therapy for Refractory Systemic Lupus Erythematosus (IC19 CAR-T).
10. Clinical Trial of CD19 Targeted CAR-T Cell in Refractory Adult SLE.
11. Chimeric Antigen Receptors T Cells for Refractory/Recurrent Lupus Nephritis in Children (CAR-T).
12. A Study to Assess CLBR001+SWI019 in Subjects With Autoimmune Diseases.
13. A Study of Rapcabtagene Autoleucel in Systemic Lupus Erythematosus (SLE) Patients With Active, Refractory Lupus Nephritis (LN).
14. Clinical Study on Targeted CD19CAR-T Therapy for Autoimmune Diseases.
15. A Phase 1 Study of SYNCAR-001 + STK-009 Without Conditioning Chemotherapy (Lymphodepletion) in Subjects With Severe, Refractory Systemic Autoimmune Rheumatic Disease.
16. IM19 CAR-T Cell Therapy in Refractory Systemic Lupus Erythematosus (SLE).
17. REACT-01: Reversing Autoimmunity Through Cell Therapy.
18. The Safety and Efficacy of Anti-CD19 CAR-T Cells in Patients With Relapsed/Refractory Autoimmune Diseases.
19. Preliminary Clinical Study of UTAA09 Injection in the Treatment of Relapsed/ Refractory Autoimmune Diseases.
20. A Study to Evaluate the Safety and Efficacy of ATHENA CAR-T in Subjects With Systemic Lupus Erythematosus.
21. KYSA-3: A Study of Anti-CD19 Chimeric Antigen Receptor T-Cell (CD19 CAR T) Therapy, in Subjects With Refractory Lupus Nephritis.
22. A Study of RJMty19 in Refractory Systemic Lupus Erythematosus (SLE).
23. A Study of CD19 Targeted CAR T Cell Therapy in Patients With Severe, Refractory Systemic Lupus Erythematosus (SLE) (CARLYSE).
24. Exploratory Clinical Study of CNCT19 Anti CD19 Cell Therapy in the Treatment of Refractory Autoimmune Diseases.
25. Anti-CD19 CAR-T Cell Therapy in Participants With Moderate to Severe Active Systemic Lupus Erythematosus.
26. A Phase 1 Study of FT819 in B-Cell Mediated Autoimmune Disease.
27. Relma-Cel for Moderate to Severe Active Systemic Lupus Erythematosus.
28. Study Evaluating SC291 in Subjects With Severe r/r B-Cell Mediated Autoimmune Diseases (GLEAM).
29. Study of Therapeutic Efficacy of Anti-CD19 CAR-T Cells in Refractory Systemic Lupus Erythematosus.
30. MB-CART19.1 in Refractory SLE.
31. Safety of PiggyBac Transposon CAR T-Cells Targeting CD-19 in Refractory Lupus.

32. RESET-SLE: A Phase 1/2 Open-Label Study to Evaluate the Safety and Efficacy of CABA-201 in Subjects With Active Systemic Lupus Erythematosus.
33. A Clinical Study of CD19 CAR-T in Active Systemic Lupus Erythematosus.
34. A Clinical Study of CD19 Universal CAR-ΓδT Cells in Active Systemic Lupus Erythematosus.
35. Universal CAR-T Cells (BRL-301) in Refractory Systemic Lupus Erythematosus.
36. KYSA-1: A Study of Anti-CD19 Chimeric Antigen Receptor T-Cell (CD19 CAR T) Therapy, in Subjects With Refractory Lupus Nephritis.
37. CNCT19 Cell Injection for Refractory Systemic Lupus Erythematosus.
38. A Study of CC-97540, CD-19-Targeted Nex-T CAR T Cells, in Participants With Severe, Refractory Autoimmune Diseases (Breakfree-1).
39. Universal CAR-T Cells (BRL-301) in Relapse or Refractory Autoimmune Diseases.
40. Safety and Efficacy of Universal CD19-Targeting CAR-ΓδT Cells in Refractory Autoimmune Diseases.
41. Safety and Efficacy of Universal CAR-T Cells (UWD-CD19) Combined with Immunosuppressants in the Treatment of Refractory Autoimmune Diseases.
42. Comparison of B-Cell Depletion by Rituximab and Anti-CD 19 CAR-T Therapy in Patients With Rheumatoid Arthritis (COMPARE).
43. Early Clinical Study of UTAA09 Injection in the Treatment of Relapsed/Refractory Autoimmune Diseases.
44. Safety and Efficacy of CD19 Targeted CAR-T Therapy for Refractory Autoimmune Disease.
45. Relmacabtagene Autoleucel for the Treatment of Systemic Sclerosis.
46. KYSA-5: A Study of Anti-CD19 Chimeric Antigen Receptor T-Cell (CD19 CAR T) Therapy, in Subjects With Systemic Sclerosis.
47. RESET-SSc: An Open-Label Study to Evaluate the Safety and Efficacy of CABA-201, a CD19-CAR T Cell Therapy, in Subjects With Systemic Sclerosis.
48. Clinical Study of CD19 CAR-T in the Treatment of Refractory Systemic Sclerosis.
49. A Study of RD06-04 in Patients With Active Autoimmune Diseases.
50. Clinical Study of CD19 Targeted Universal Chimeric Antigen Receptor T Lymphocytes (UCAR-T) for the Treatment of Refractory Juvenile Dermatomyositis (RJDM).
51. Clinical Trial of CD19-Targeted CAR-T Therapy for Refractory Juvenile Dermatomyositis.
52. Study of KYV-101 Anti-CD19 CAR T Therapy in Adult Dermatomyositis.
53. RESET-Myositis: An Open-Label Study to Evaluate the Safety and Efficacy of CABA-201 in Subjects With Active Idiopathic Inflammatory Myopathy or Juvenile Idiopathic Inflammatory Myopathy.
54. Phase 2 Study Evaluating Rapcabtagene Autoleucel in Participants With Severe Active GPA or MPA.
55. Anti-CD 19 CAR-T Cell Therapy in Patients with ANCA Vasculitis (IDEAL).
56. Study of Therapeutic Efficacy of Anti-CD19 CAR-T Cells in Children With Refractory Refractory AAV.
57. CAR-T Cells in Systemic B Cell Mediated Autoimmune Disease (CASTLE).
58. Phase 2 Study of Rapcabtagene Autoleucel in Myositis.
59. A Study of SYS6020 Injection in Refractory Active Systemic Lupus Erythematosus.
60. Equecabtagene Autoleucel Injection (Eque-Cel) for Relapsed/Refractory Systemic Lupus Erythematosus (SLE).
61. Safety and Efficacy of PRG-1801 for Refractory Lupus Nephritis and IgG4-Related Disease.

62. BAFF CAR-T Cells (LMY-920) for Systemic Lupus Erythematosus.
63. Refractory ANCA Associated Vasculitis and Lupus Nephritis Treated With BCMA-Targeting CAR-T Cells.
64. Descartes-08 for Patients With Systemic Lupus Erythematosus (SLE-001).
