# Supplementary material for: Chimeric Antigen Receptor T Cell Immunotherapy for Autoimmune Rheumatic Disorders: Where Are We Now?
Source: Cells. 2025 Aug 12;14(16):1242. doi: 10.3390/cells14161242 (PMC12384554; doi:10.3390/cells14161242)
Supplement: Supplementary file 1 [file cells-14-01242-s001.zip › cells-3781980_TableS2.pdf]

Table S2. A summary of the ongoing clinical trials on the role of anti-BCMA CAR-T cell products in autoimmune rheumatic disorders.

Clinical trial registration number, Reference	Country	Design-Phase	CAR-T cell product	Autoimmune rheumatic disease	Primary study endpoints	Status
NCT06694298 [1]	China	Phase I	SYS6020 (anti-BCMA)	Refractory SLE	-Type and incidence of DLT<br>-Incidence of TEAEs<br>-RD<br>-Proportion of patients who achieved SLE remission	Not yet recruiting
NCT06902844 [2]	China	Open label, single-arm, phase NS	Equecabtagene autoleucel (anti-BCMA)	Relapsed/ refractory SLE	Response rate 4-6 months post treatment	Recruiting
NCT06497387 [3]	China	Open label, single-arm, phase I	PRG-1801 (anti-BCMA)	-Refractory SLE-LN<br>-IgG4-related disease	-Incidence of TEASs<br>-Safe CAR-T infusion dose	Recruiting
NCT06340750 [4]		Open label, single-arm, phase I	LMY-920 (anti-BAFF)	SLE	-Incidence and type of TEASs<br>-Phase II RD	Not yet recruiting
NCT06277427 [5]	China	Open label, multiple arm, phase I	PRG-1801 (anti-BCMA)	-SLE-LN<br>-AAV	- Incidence and type of TEASs<br>- Incidence of DLTs	Recruiting
NCT06038474 [6]	United States	Open label, single arm, phase II	Descartes-08 (anti-BCMA)	SLE	Incidence and type of TEASs	Recruiting

AAV: ANCA-associated vasculitis, ANCA: anti-neutrophil cytoplasmic antibodies, BCMA: B Cell maturation antigen, CAR-T: chimeric antigen receptor T-cell therapy, DLT: dose-limiting toxicity, RD: rheumatic disease, SLE: systemic lupus erythematosus, TEAEs: treatment-emergent adverse events.

References

1. A Study of SYS6020 Injection in Refractory Active Systemic Lupus Erythematosus.
2. Equecabtagene Autoleucel Injection (Eque-Cel) for Relapsed/Refractory Systemic Lupus Erythematosus (SLE).
3. Safety and Efficacy of PRG-1801 for Refractory Lupus Nephritis and IgG4-Related Disease.
4. BAFF CAR-T Cells (LMY-920) for Systemic Lupus Erythematosus.

5. Refractory ANCA Associated Vasculitis and Lupus Nephritis Treated With BCMA-Targeting CAR-T Cells.
6. Descartes-08 for Patients With Systemic Lupus Erythematosus (SLE-001).
