# Supplementary material for: Chimeric Antigen Receptor T Cell Immunotherapy for Autoimmune Rheumatic Disorders: Where Are We Now?
Source: Cells. 2025 Aug 12;14(16):1242. doi: 10.3390/cells14161242 (PMC12384554; doi:10.3390/cells14161242)
Supplement: Supplementary file 1 [file cells-14-01242-s001.zip › cells-3781980_TableS3.pdf]

Table S3. A summary of the ongoing clinical trials on the role of bispecific and trispecific CAR-T cell products in autoimmune rheumatic disorders

Clinical trial registration number, Reference	Country	Design-Phase	CAR-T cell product	Autoimmune rheumatic disease	Primary study endpoints	Status
NCT06947460 [98]	China	Open-label, single-arm, Phase I-II	Bispecific anti-CD19/ BCMA	-Refractory SLE-LN<br>-SSc<br>-pSS with PAH	Type and incidence of DLT within 28 days after infusion	Not yet recruiting
NCT06653556 [90]	China	Single-arm, open-label, Phase I	LCAR-AIO (trispecific anti-CD19/CD20/ CD22)	Relapsed/ refractory SLE	-Incidence of DLT<br>-Incidence, severity, and type of TEAEs	Recruiting
NCT06866080 [91]	China	Single-arm, open-label, early Phase I	LCAR-AIO (trispecific anti-CD19/CD20/ CD22)	Relapsed/ refractory<br>-SLE<br>-AAV<br>-SSc<br>-IIM	Proportion of patients who achieved disease remission	Recruiting
NCT06708845 [99]	Germany	Open-label, non-randomized, phase I	Zamto-cel (bispecific anti-CD19/ CD20)	-SLE<br>-SLE-LN<br>-SSc/ dcSSc	- Type and incidence of DLT within 28 days after infusion<br>-Incidence of TEAEs<br>-RD for phase II trial	Not yet recruiting
NCT06785519 [100]	China	Open label, single-arm, early phase I	Bispecific anti-CD19/ BCMA	Refractory/ relapsed SLE LN	-Incidence of TEAEs<br>-DLT	Recruiting
NCT06794008 [101]	China	Open label, single-arm, phase II	Bispecific anti-CD19/ BCMA	-SLE<br>-pSS<br>-IIM<br>-SSc<br>-Behcet's disease<br>-AAV<br>-IgG4-related disease<br>-APS<br>-Acquired TTP	ORR	Recruiting
NCT06897930 [102]	NS	Open label, single-arm, phase Ib/ II	AZD0120 (bispecific anti -CD19/ BCMA)	Refractory SLE	-Incidence of TEAEs<br>-RD for phase II trial<br>-Response to treatment	Not yet recruiting

Table S3. A summary of the ongoing clinical trials on the role of bispecific and trispecific CAR-T cell products in autoimmune rheumatic disorders

NCT06920433 [103]	China	Open label, single-arm, early phase I	Universal allogeneic (bispecific anti -CD19/ BCMA)	Relapsed/ refractory SLE	-DLT<br>- Incidence of TEAEs<br>-Clinical response	Not yet recruiting
NCT06934447 [92]	China	Open label, single-arm, phase I	Bispecific anti -CD70/ BCMA	Refractory SLE (pediatric patients)	- Incidence of TEAEs<br>-Clinical response	Recruiting
NCT06935474 [104]	United States	Open label, multiple arm, phase I/ II	C-CAR168 (bispecific anti -CD20/ BCMA)	Refractory:<br>-SLE<br>-SLE-LN	- Incidence of TEAEs<br>-DLT	Not yet recruiting
NCT06567080 [105]	China	Open label, single-arm, phase I	JWCAR201 (Bispecific anti-CD19/CD20)	-Relapsed/ refractory LBCL<br>-SLE	-Incidence of TEASs<br>-DLT	Not yet recruiting
NCT06530849 [106]	China	Open label, single-arm, phase I/ II	GC012F (bispecific anti CD19/ BCMA)	Refractory SLE	-DLT<br>-Clinical response	Recruiting
NCT06503224 [107]	China	Open label, single-arm, phase not applicable	SCAR02 (bispecific anti CD19/ BCMA)	Refractory<br>-RA<br>-SLE<br>-pSS<br>-SSc	Incidence of TEASs	Recruiting
NCT06497361 [108]	China	Open label, single-arm, early phase I	PRG-2311 (bispecific anti-CD19/ BCMA)	-Refractory SLE-LN<br>-IgG4-related disease	-Incidence of TEASs<br>-Safe CAR-T infusion dose	Recruiting
NCT06462144 [109]	China	Open label, single-arm, early phase I	IMPT-514 (bispecific anti-CD19/ BCMA)	-Active/ Refractory SLE<br>-AAV<br>-IIM	-Incidence of TEASs<br>-DLT	Recruiting
NCT06428188 [110]	China	Open label, single-arm, phase I/ II	Bispecific anti-CD19/ BCMA	-SLE<br>-IIM<br>-SSc<br>-IGG4-associated disease<br>-pSS<br>-RA<br>-Connective tissue disease-associated interstitial lung disease<br>-ITP	-Incidence and severity of DLTs	Recruiting

Table S3. A summary of the ongoing clinical trials on the role of bispecific and trispecific CAR-T cell products in autoimmune rheumatic disorders

				-PBC		
NCT06373081 [111]	China	Open label, single-arm, phase not applicable	Bispecific anti-CD19/ CD3E	Relapsed/ refractory -SLE -pSS -SSc -IIM -AAV -catastrophic APS	-DLT -Clinical response	Recruiting
NCT06350110 [112]	China	Open label, single-arm, phase I/ II	BH002 injection (bispecific anti-CD19/ BCMA)	Relapsed/ refractory SLE	Incidence and severity of DLTs	Recruiting
NCT06349343 [113]	China	Open label, single-arm, phase I	Bispecific anti-CD19/ BCMA	Refractory/ Moderate-to-severe SLE	Incidence and type of TEASs	Recruiting
NCT06285279 [97]	China	Open label, single arm, phase I	FKC288 (bispecific anti-CD19/ BCMA)	-SLE-LN -AAV - IGG4-associated disease -Membranous nephropathy	- Incidence and type of TEASs - Incidence of DLTs	Recruiting
NCT06249438 [93]	China	Open label, single arm, phase I	C-CAR168 (bispecific anti-CD20/ BCMA)	-SLE -IMNM -NMO -RRMS - Myasthenia Gravis	- Incidence and type of TEASs - Incidence of DLTs	Recruiting
NCT05858684 [114]	China	Open label, single arm, early phase I	GC012F (bispecific anti-CD19/ BCMA)	Refractory SLE	- Incidence of DLTs - Incidence and type of TEASs	Unknown status
NCT06947473 [115]	China	Open label, single arm, phase I	Allogeneic umbilical cord blood (bispecific anti-CD19/ BCMA)	-SLE-LN -SSc -pSS	- Incidence of DLTs - Incidence and type of TEASs	Not yet recruiting
NCT06548607 [116]	China	Open label, single arm, early phase I	RD06-04 (anti-CD19), RD06-05 (bispecific anti-CD19/ BCMA)	Active -SLE -SSc -AAV -IIM -pSS	Incidence and type of TEASs	Recruiting

Table S3. A summary of the ongoing clinical trials on the role of bispecific and trispecific CAR-T cell products in autoimmune rheumatic disorders

NCT05085431 [117]	China	Open label, single arm, early phase I	Bispecific anti-CD19/ BCMA	Refractory pSS	- Incidence of DLTs<br>- Incidence and type of TEASs	Unknown status
NCT06941129 [118]	China	Open label, single arm, phase I	Universal allogeneic bispecific anti-CD19/ BCMA	Relapsed/ refractory:<br>-SLE<br>-SSc<br>-IIM<br>-AAV	- Incidence of DLTs<br>- Incidence and type of TEASs<br>-Clinical efficacy	Recruiting

AAV: ANCA-associated vasculitis, ANCA: anti-neutrophil cytoplasmic antibodies, APS: antiphospholipid syndrome, BCMA: b cell maturation antigen, CAR-T: chimeric antigen receptor T-cell therapy, dcSSc: diffuse cutaneous systemic sclerosis, DLT: dose-limiting toxicity, IIM: idiopathic inflammatory myopathies, ITP: immune thrombocytopenia, LBCL: large b-cell lymphoma, NMO: neuromyelitis optica, NS: not specified, ORR: overall response rate, PAH: pulmonary arterial hypertension, PBC: primary biliary cholangitis, pSS: primary Sjogren’s syndrome, RA: rheumatoid arthritis, RD: recommend dose, RRMS: relapsing-remitting multiple sclerosis, SLE: systemic lupus erythematosus, SLE-LN: systemic lupus erythematosus–lupus nephritis, SSc: systemic sclerosis, TEAEs: treatment-emergent adverse events.
